# Advances in adjunct therapy against tuberculosis: Deciphering the emerging role of phytochemicals

**DOI:** 10.1002/mco2.82

**Published:** 2021-08-05

**Authors:** Samreen Fatima, Anjna Kumari, Ved Prakash Dwivedi

**Affiliations:** ^1^ Immunobiology Group International Centre for Genetic Engineering and Biotechnology New Delhi India

**Keywords:** adjunct therapy, cytokines, immunomodulation, *Mycobacterium tuberculosis*, phytochemicals, T cells

## Abstract

Eastern countries are a major source of medicinal plants, which set up a rich source of ethnopharmacologically known medicines used in the treatment of various diseases. These traditional medicines have been known as complementary, alternative, or nonconventional therapy across globe for ages. Tuberculosis (TB) poses a huge global burden and leads to maximum number of deaths due to an infectious agent. Treatment of TB using Directly Observed Treatment Short‐course (DOTS) therapy comprises multiple antibiotics is quite lengthy and causes serious side‐effects in different organs. The length of the TB treatment leads to withdrawal from the patients, which paves the way for the emergence of drug resistance in the bacterial population. These concerns related to therapy need serious and immediate interventions. Traditional medicines using phytochemicals has shown to provide tremendous potential in TB treatment, mainly in the eradication of *Mycobacterium tuberculosis* (*M.tb*), increasing natural immunity, and managing the side effects of anti‐TB drugs. This review describes the antituberculosis potential of selected ethnopharmacologically important phytochemicals as potential immune‐modulator and as an adjunct‐therapy in TB. This review will be a useful reference for researchers working on ethnopharmacology and will open the door for the discovery of novel agents as an adjunct‐therapy to tuberculosis.

## Graphical abstract

The different modes of action of phytochemicals in adjunct therapy for the treatment of TB.

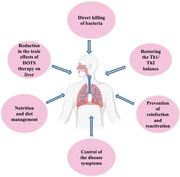

## 1 INTRODUCTION

Tuberculosis (TB) is a communicable disease caused by slow‐growing, acid‐fast bacillus *Mycobacterium tuberculosis (M. tuberculosis)*. BCG (*Bacillus Calmette‐Guérin*) is the only validated vaccine against pulmonary TB. However, despite years of vaccination and antibiotic therapy, TB remains a threat with 10.5 million new cases reported in 2019.^1^ WHO reports that there are documented 10.5 million new cases of TB each year with almost 1.8 million deaths. Needless to say that about one‐third of the cases still remain unreported.^2^

**FIGURE 1:**
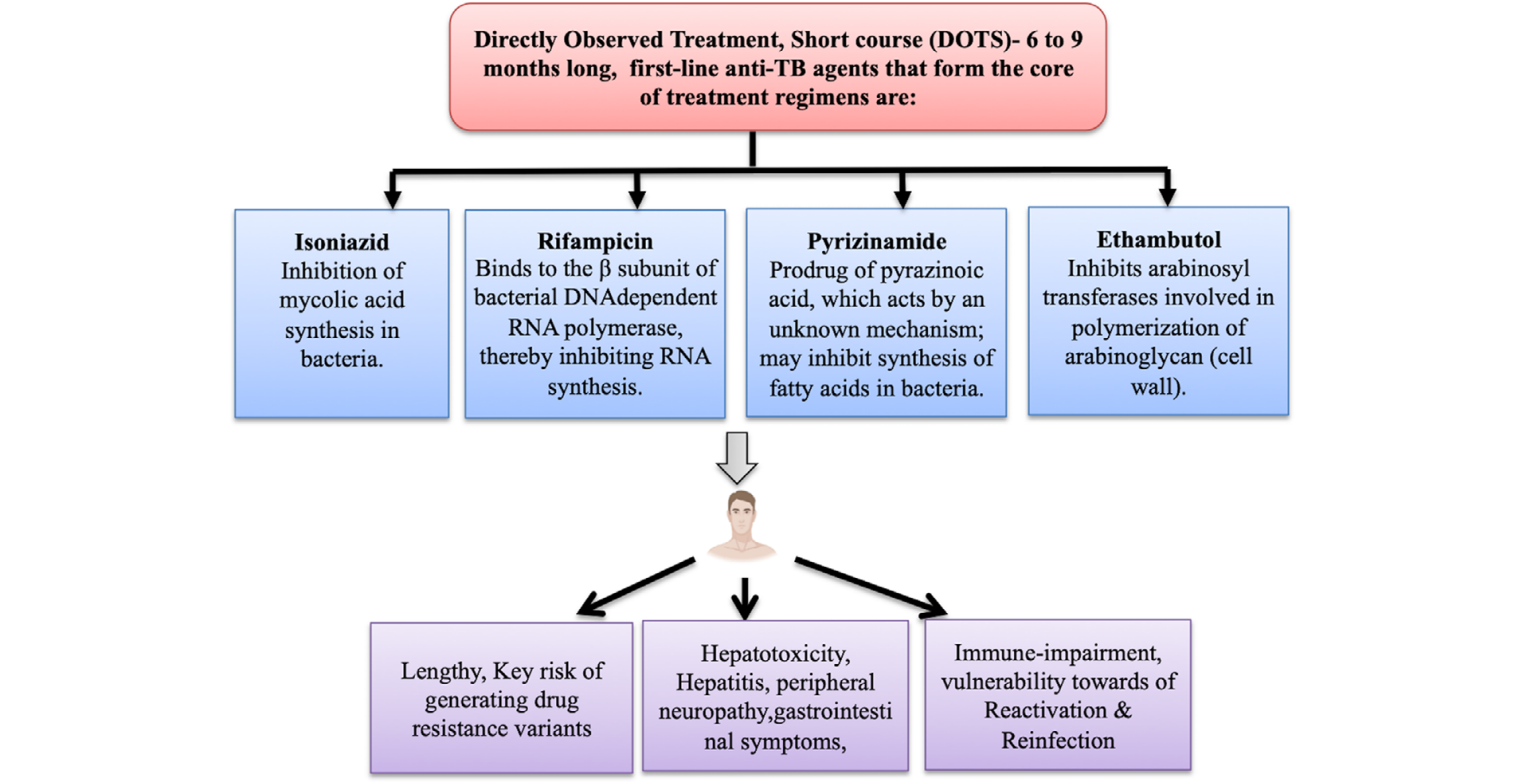
Tuberculosis treatment and associated side effects

*M. tuberculosis* is inhaled in the form of small aerosol droplets containing the bacilli and is transmitted to healthy individual from an infected person through the respiratory route via inhalation into the lungs. The bacteria travel through the lungs and reside in the alveoli of the lungs. In 90% of the infected individuals, the infection does not lead to active disease and is called a latent state of infection where bacteria can live for many years in a nonreplicating state. In the remaining 10% individuals, who are in immune‐compromised state, the disease may take the active replicating form.^3^ Treatment of TB is globally known as “Directly Observed Treatment Short‐course (DOTS).” It is a multidrug and a long‐term therapy. It consists of many antibiotics that cause severe toxicity and side effects.^4^, ^5^ After approximately 4 weeks of treatment with antibiotics, the patient's condition improves significantly, causing him to discontinue the treatment regime, which can give rise to drug‐resistant persister populations. Moreover, the treatment is expensive considering the income of the patients in the developing countries and there is a significant risk of the generation of multidrug‐resistant (MDR) and extensive drug‐resistant (XDR) strains of bacteria.^4^, ^5^ In some adverse cases, the side effects of the antibiotics cause liver diseases such as hepatitis with a mortality of 5% and dampens the hosts’ T‐cell immune response. Frontline anti‐TB drugs such as rifampin have adverse side effects like thrombocytopenia and itching, isoniazid causes neuropathy and T‐cell reduction. Isoniazid, pyrazinamide, and rifampin cause drug‐induced hepatitis with high mortality rate.^5^ The side effects of the DOTS therapy have been schematically represented in Figure 1. Therefore, we need an alternative therapeutic approach that may limit the side‐effects associated with anti‐TB therapy.

**FIGURE 2:**
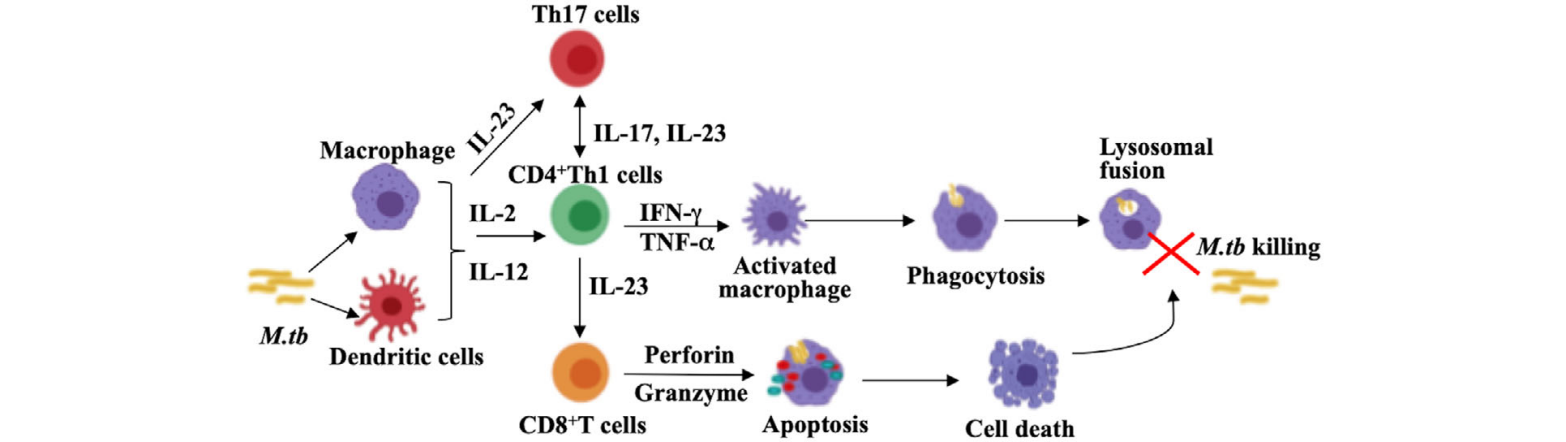
The role of T cells in Tuberculosis pathogenesis and bacterial clearance

A thorough understanding of the host immune response after *M. tuberculosis* infection is essential for the development of effective vaccine strategies and immune therapeutics. *M. tuberculosis* infects the healthy individuals by getting inhaled in form of the bacilli containing aerosol droplets, which are released from an infected person. After getting inhaled, the bacteria travel through the mucosal pathway and reach the alveoli of the lungs. The alveolar macrophages are the first cells that encounter these pathogens and engulf them.^6^ These macrophages secrete cytokines and chemokines that recruit other inflammatory cells to the site of infection. Alveolar macrophages together with neutrophils and other inflammatory cells organize themselves into compact structures called granulomas. Granulomas form in the lungs to limit the growth of *M. tuberculosis*. The macrophages and dendritic cells migrate to lymph nodes and present the mycobacterial antigens to the T cells in the lymph nodes. Upon antigen presentation, naïve T cells differentiate into CD4+ and CD8+ T cells and move back to the lungs.^6^ These T cells mainly T‐helper 1 (Th1) cells and T‐helper 17 (Th17) cells eliminate the bacteria by secretion of pro‐inflammatory cytokines such as IFN‐γ, TNF‐α, and cytolytic killing mechanisms, respectively. The granuloma consisting of a core of infected macrophages, surrounded by epithelioid cells, lymphocytes, neutrophils, and mesenchymal stem cells (MSCs) is maintained by a delayed‐type hypersensitivity (DTH) response to the bacterial antigens and tumor necrosis factor‐alpha (TNF‐α).^6^, ^7^ In 90% of the infected individuals, the *M. tuberculosis* remains in the granulomatous structure for a very long time in a nonreplicating, asymptomatic state called as the “dormant state.”^7^ Antigen‐specific regulatory T (Treg) cells and T helper 2 (Th 2) cells also get differentiated and counter the pro‐inflammatory response in order to maintain homeostasis in the infected host.^8^ *M. tuberculosis* resides in the granuloma in dormant state for decades by utilizing the host lipids and slowing down their replicative genes^9^ and wait for an opportunity such as a weakened immune system to reactivate and cause active disease.^8^ Elimination of actively dividing bacteria in the macrophages involves the role of macrophages and T helper (Th) cell responses whereas latent *M. tuberculosis* that is supported by the host MSCs requires myriad other factors and responses to get cleared up.^9^ Thus, Th1 and Th17 cells are the most important arms of adaptive immunity that are responsible for maximum protection against tuberculosis infection.^10^, ^11^ The crucial role of T cells and their secreted cytokines in TB pathogenesis has been described in Figure 2.

**FIGURE 3:**
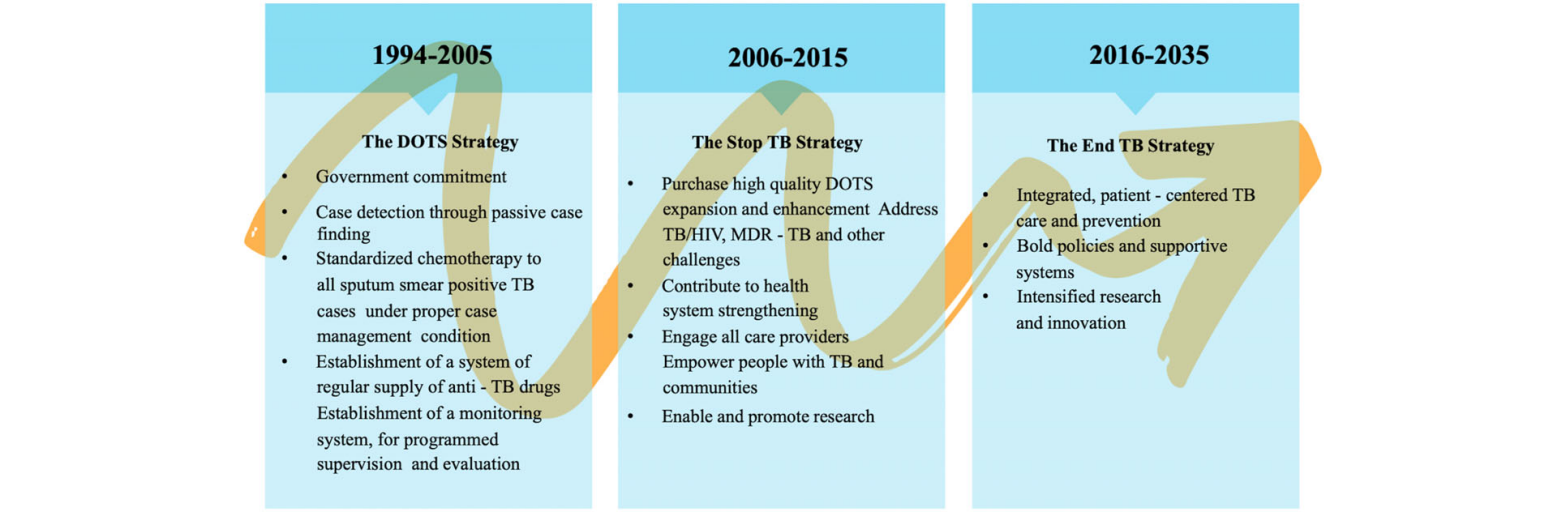
Evolution and the pillars of global TB eradication strategies

The major challenges faced by TB eradication programmes are the failure of BCG vaccination in protection against adult pulmonary TB, the length and side effects of DOTS therapy, slow progress in new drug development approaches, and the emergence of drug resistance in TB strains.^1^, ^5^ Therefore, we urgently need an improved alternate therapeutic approach that can overcome these limitations to deal with the deadly pathogen better. This is the utmost requirement of the 2035 end TB goal as set by the WHO. We have described the pillars of global TB management programme in Figure 3. Thus, in such a scenario a therapy that could be derived from natural plants to balance out the toxicity associated with the drugs used in the DOTS therapy and thereby rejuvenates the immune system, to avoid or prevent disease reactivation is urgently needed and would be very beneficial. These compounds are mainly extracted from the plants that have been used in traditional medicines for ages and have shown promising effects. These compounds may be used either as drug candidates or immunomodulatory agents with therapeutic potential against TB. Here, in this review paper, we would mainly focus on ethnomedical agents/compounds, which have been used as immunomodulators or as an adjunct therapy to reduce the toxicity of the DOTS therapy and induce Th1 response and/or Th17 host protective immune response simultaneously. This review is an effort to summarize such compounds, known as phytochemicals with their detailed mechanism of action.

## 2 TREATMENT OF TUBERCULOSIS USING TRADITIONAL MEDICINES DERIVED FROM PLANTS

Tuberculosis is an ancient disease as evident from the skeletal deformities found in the Egyptian mummies belonging to 2400 BC, but it has been only around 2000 years since the first report of TB was documented in India and China.^12^ The traditional treatment of TB is practiced based on indigenous knowledge possessed by the local healers. Earliest known treatment of this disease consisted of natural methods to manage TB and comprised various rituals derived from old traditional practices.^13^ Age‐old practices related to TB treatment that have been handed down from generation to generation and are still part of TB therapy in various countries of South Africa and Asia.^14^, ^15^, ^16^, ^17^, ^18^, ^19^, ^20^, ^21^ These traditional therapies although are not capable of completely eradicating the disease but are quite effective in treating the respiratory disorders associated with TB and reducing the toxicity associated with antituberculosis therapy (ATT) as observed by the treatment provided in the patients by the traditional healers. Researchers all over the globe are trying to find new antimicrobials from rich and medically significant plant secondary metabolites belonging to the Indian subcontinent, South Africa, and other eastern countries.^15^, ^16^, ^18^

Since traditional treatment is practiced mostly on a local scale therefore, the plants used for the treatment vary considerably based on the country or place where it is followed. Researchers from high TB burden countries have reported different plant species used locally by traditional healers for the treatment of TB and as the literature suggests, most widely used plant species in the treatment of TB belong to Asteraceae, Asparagaceae, Amaryllidaceae, Apiaceae, Rutaceae, Solanaceae, and Leguminosae families where plant parts such as roots, leaves, barks bulbs, and fruits contribute to the treatment of the patients.^20^, ^22^, ^23^, ^24^

These plant extracts do not eradicate the bacteria all on their own but they seem to play a very crucial role in managing symptoms related to TB such as prolonged cough, chest pains, fatigue, appetite loss, and fever that increase the level of discomfort in patients. These plant extracts or secondary metabolites exert expectorant, bronchodilator, anti‐inflammatory, and antipyretic effects. Since hundred years, plants such as *Tussilago farfara* and *Pulmonaria officinals* are being explored for these properties in the treatment of TB.^25^ Treatment of TB through the use of plants/phytochemicals constitutes a comprehensive approach, which could give TB treatment an improved outlook.

## 3 PHYTOCHEMICALS USED IN TUBERCULOSIS THERAPY WITH THEIR POTENTIAL ROLE IN ATT

The word “Phytochemicals” refers to a variety of naturally occurring and biologically active substances in plants that have protective or disease curing properties. The word “phytochemical” has been derived from the Greek word “phyton,” which means plants.^26^ The phytochemicals are basically the chemicals produced in plants mostly as part of protection mechanism utilized by the plants to help them thrive predators and pathogens. They are the product of primary or secondary metabolism of the plant.^26^ These compounds have a history of being used in biomedical therapies and their therapeutic properties are mainly associated with the presence of many different compounds such as carotenoids, flavonoids, isothiocyanates, indoles, monoterpenes, and phenolic acids.^27^ The British Nutrition Foundation has classified these phytochemicals into four major categories: terpenoids, phenolics, nitrogen‐containing compounds, and sulfur‐containing compounds.^27^ Till now, an ample number of phytochemicals have been screened but due to the lack of detailed analysis, this area of research has been continuously neglected and therefore needs to be strengthened by new research. Limited studies have been conducted so far in exploring the potential role of phytochemicals in antituberculosis therapy. Many researchers now giving attention to the role of phytochemicals in TB therapy, as this therapy might help improve the effects of DOTS treatment. Some studies report the promising effect of the phytochemicals against *M. tuberculosis* bacteria.^28^, ^29^ However, these studies fail to compile the host protective mechanisms or the immunotherapeutic use of these compounds. The domain of phytochemical studies against *M. tuberculosis* is very broad and promising and is therefore in drastic need of further exploration. We know that DOTS therapy while eliminating the bacteria dampens the host immune system. An adjunct drug or compound that could prevent the dampening of the immune cells will prove to be a boon for TB treatment.

In the following section, we have tried to summarize the known phytochemicals, which have been used in TB therapy. We have also discussed the antimicrobial activity of these phytochemicals with special impetus on their use in the prevention and treatment of tuberculosis.

### 3.1 Allicin

**FIGURE 4:**
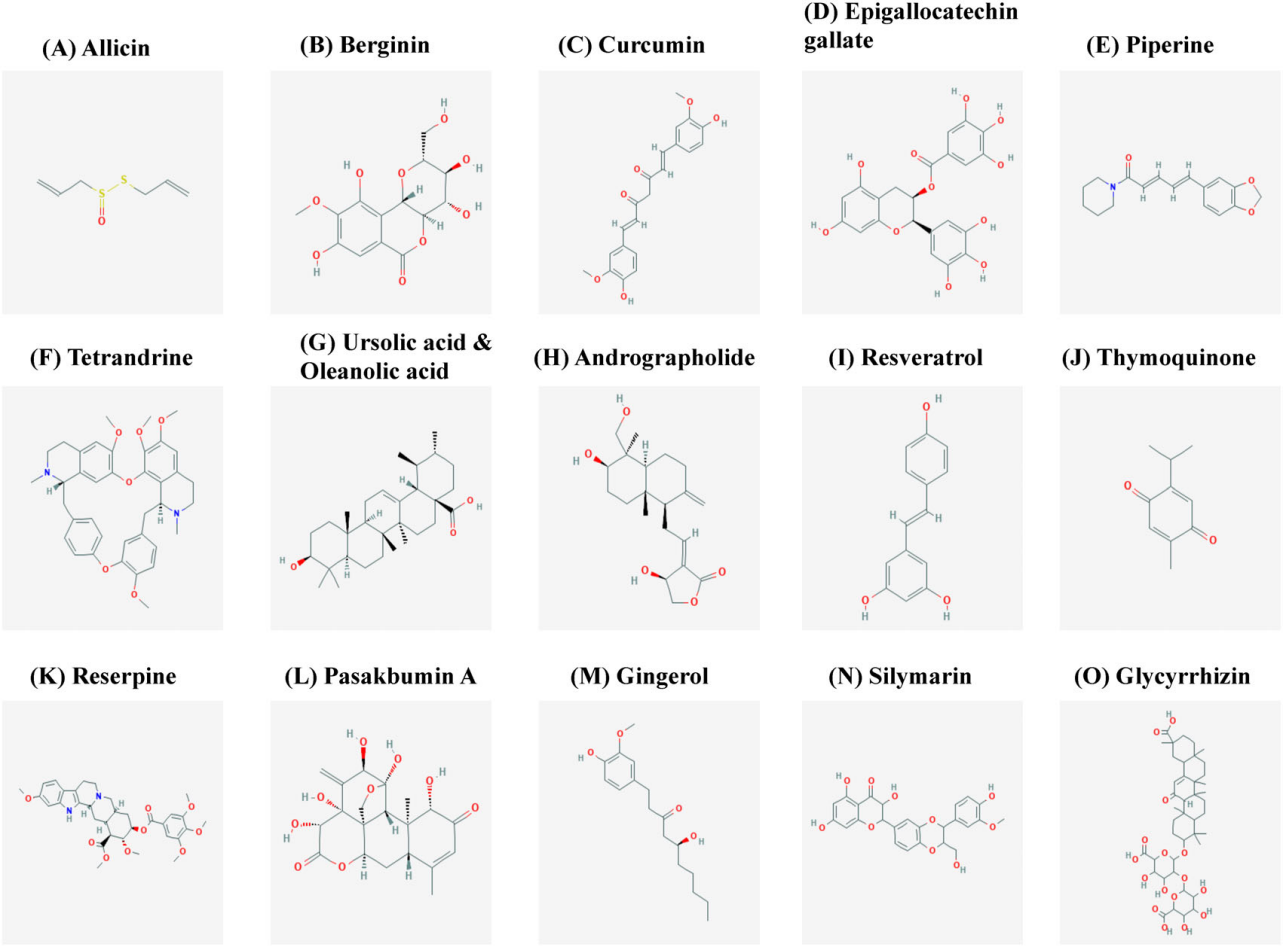
Structure of phytochemicals used for the treatment of tuberculosis. (A) Allicin, (B) Bergenin, (C) Curcumin, (D) Epigallocatechin gallate, (E) Piperine, (F) Tetrandrine, (G) Ursolic acid and Oleanolic acid, (H) Andrographolide, (I) Resveratrol, (J) Thymoquinone, (K) Reserpine, (L) Pasakbumin A, (M) Gingerol, (N) Silymarin, and (O) Glycyrrhizin (Adapted from PubChem)

Garlic (*Allium sativum*) is a commonly used food ingredient that has been widely acclaimed for its contribution to human health for centuries. Garlic is used in the prevention and treatment of a variety of infectious and noninfectious diseases.^30^, ^31^, ^32^ Garlic is a strong antibacterial agent and can inhibit the growth of both Gram‐positive and Gram‐negative bacteria.^33^ Allicin is the main constituent of garlic with potential antimicrobial properties (Figure 4A). It is an oxygenated sulfur compound, which is chemically known as thio‐2‐propene‐1‐sulfinic acid S‐allyl ester. Allicin is an inhibitor of sulfhydryl metabolic enzymes. It interacts with the SH‐ group of the enzymes to exert its antimicrobial effects.^34^ Garlic has inexhaustible research history against mycobacterial infection. In 1944, Rao et al reported the reduction in *M. tuberculosis* replication upon treatment with allicin. They observed a bacteriostatic effect at low concentrations and a bactericidal effect at higher concentrations of the compound.^35^ A further study by Ratnakar et al in 1995 reported that garlic was able to inhibit the growth of isoniazid‐susceptible and ‐resistant strains, H37Rv and TRC‐C1193, respectively.^36^ In the following years, it was reported that allicin reduces *M. tuberculosis* burden in the host by increasing the activity of the enzyme, glutathione peroxidase.^37^ Another interesting in vitro study compared the antitubercular activity of *Allium sativum* with standard antibiotics using the disc diffusion method.^38^ These results established that garlic exhibited maximal activity against multiple drug‐resistant *M. tuberculosis* as well.^38^ In 2006, Hasan et al studied the reduction in Reactive Oxygen Species (ROS) expression induced by the bacilli, upon treatment with allicin.^39^ In 2014, Vishwanathan et al reported in an in vitro study that garlic extract and garlic oil both show decent antimycobacterial activity when compared to standard drugs, using zone inhibition method.^40^ A recent study by Dwivedi et al has given some strong proofs to establish the therapeutic potential of allicin in the pathogenesis of tuberculosis. Allicin/garlic extract displays direct killing of Mycobacteria and leads to the induction of pro‐inflammatory cytokines in macrophages while also limiting *M. tuberculosis* infection inside the cells by interacting with the cell surface receptors responsible for *M. tuberculosis* entry.^41^ Experiments done in mice model establish that treatment of infected mice with allicin/garlic extract leads to a significant reduction in bacterial burden mainly due to host protective Th1 response, which eliminates the pathogens in a much lesser time duration compared to the conventional treatment coarse. Furthermore, garlic extract also has been shown to reverse the immune‐dampening effects associated with the use of standard TB drugs.^41^ In a nutshell, allicin/garlic extract showed very promising results in infected mice when used alone or as an adjunct to classical antibiotics for both drug‐sensitive and ‐resistant strains. Another compound derived from garlic, known as ajoene, has shown tremendous effectiveness in TB treatment due to its ability to induce autophagy and ROS synthesis.^40^, ^41^, ^42^ The therapeutic value of these compounds and their broad‐spectrum antimicrobial activity suggests garlic and its derivatives can be a beneficial addition to ATT.

### 3.2 Bergenin

Bergenin is a natural secondary metabolite found in different parts of several plants.^43^ It is also known as cuscutin and is a trihydroxybenzoic acid glycoside (Figure 4B). It is one of the active phytochemicals in herbal and ayurvedic formulations. Bergenin has a myriad of ethanomedical properties like antibacterial, antiviral, antifungal, antitussive, anti‐inflammatory, antitumor, antidiabetic, and wound‐healing properties.^44^ Of late, the effectiveness and properties of bergenin have been studied by different groups in different disease contexts. Nunomora et al did some initial research on the existing anti‐inflammatory properties of bergenin.^45^ Later, in 2016, Khan et al reported that bergenin has remarkable activity against the chloroquine‐sensitive *Plasmodium falciparum* and also shows antioxidant properties.^46^ Recently Dwivedi et al have demonstrated the effectiveness of this phytochemical in tuberculosis management. Their investigation revealed that bergenin treatment in infected macrophages led to the activation of the MAP kinase and ERK pathways, which further lead to TNF‐α, nitric oxide (NO), and Interleukin‐12 (IL‐12) production. Further, when observed in murine model, bergenin induces the expression of Th1 and Th17 immune responses and limit the replication of the bacteria.^47^ They also studied that a combination therapy of DOTS along with bergenin reverses the immune damage and reduces the duration of clearance of *M. tuberculosis* in mice.^48^ Therefore, to summarize cotreatment with bergenin and isoniazid reduces the side effects associated with isoniazid such as immune dampening, while promoting the generation of long‐lasting, central memory T‐cell responses.^47^, ^48^ Notably, bergenin acted well in the elimination of drug‐resistant strains as well. Therefore, bergenin can be a prospective adjunct to current TB therapy.

### 3.3 Curcumin

Curcumin, also known as “Indian Yellow Gold” is a polyphenol, diferuloylmethane, which is responsible for the bright yellow‐orange color of the Indian spice turmeric (*Curcuma longa*) (Figure 4C). Turmeric is the most commonly consumed spice in India. It has been used in India and China since time immemorial, in traditional treatments and as an antibiotic.^49^ The compound “curcumin” was isolated by German scientists Vogel and Pelletier in 1815.^50^ Curcumin has numerous healing properties that have been a topic of research by scientists all over the world.^51^, ^52^ The most studied activity of curcumin in the past years are its antitumor effects and antibacterial properties.^53^ In 1949, Schraufstätter and Bernt described the bactericidal activity of curcumin against various bacteria including the TB causing pathogen, *M. tuberculosis*.^54^ The antibacterial potential of curcumin has gained tremendous attention in the past few decades owing to its extraordinary antibiotic properties.^55^, ^56^ Curcumin has anti‐inflammatory and antioxidative potential and targets several pathways important for bacterial survival.^57^

Curcumin is beneficial in the treatment of diseases such as cancer, diabetes, kidney disorder, and neurocognitive disorders by modulating the expression of Nrf2 gene. The anti‐inflammatory role of curcumin is exhibited mainly via the downregulation of NF‐κB gene signaling and its downstream genes and proteins.^58^, ^59^ In cancer, curcumin downregulates the expression and activity of cyclooxygenase (COX‐2) gene along with reduced activity of inducible nitric oxide synthase (iNOS) by suppressing the transcription factor NF‐κB. This in turn has a role in suppression of progressing tumorigenesis.^60^, ^61^ It acts effectively to eliminate *M. tuberculosis* in TB‐infected patients. Therefore, it would be very interesting to further explore the action mechanism of this phytochemical in tuberculosis treatment. Curcumin is also reported to prevent anti‐TB drug‐induced hepatic damage.^62^ It exhibits dose‐dependent inhibition of intracellular growth for *M. tuberculosis*, H37Rv.^63^ It is a potent inducer of apoptosis, which is an effector mechanism used by macrophages to kill intracellular pathogens. The antimicrobial potential of macrophages is increased upon treatment with curcumin, by an upregulation in the expression of apoptosis and autophagy genes.^64^

Although curcumin has myriad health benefits, it is relatively unstable and has poor bioavailability because of being rapidly eliminated from the body.^65^ To overcome this limitation, Baldwin et al synthesized monocarbonyl analogs of curcumin and tested for their efficiency in reducing the replication of *M. tuberculosis* and *Mycobacterium marinum* (Mm) and found a remarkable reduction in the number of Mm and *M. tuberculosis* using several analogs.^66^ This study paved the way for synthesis of more structural analogs that could interact better with the frontline anti‐TB drugs. Another modification of curcumin that is nanoparticle‐formulated curcumin was generated by Tousif et al and studied for its effect against active TB in mice model. Nanocurcumin exhibits a fivefold increase in bioavailability compared to curcumin extracted from turmeric and is around ∼200 nm in size.^67^

As is the demand of TB treatment, curcumin nanoparticles effectively reverse the hepatotoxicity conferred by antitubercular drugs and reduce the incidence of reinfection and reactivation in mice. Thus, it compensates for the major shortcoming of the DOTS therapy by shortening the time required to achieve complete clearance of the bacilli from the lung and thereby diminishing the probability of generation of drug resistance among *M. tuberculosis* strains.^67^ Therefore, an adjunct therapy comprising nanocurcumin together with DOTS therapy could be included in the treatment of tuberculosis.^67^ Lately, they further tested curcumin nanoparticles for their possible role in augmenting the effectiveness of *Mycobacterium bovis* (BCG) vaccine. They found that curcumin nanoparticles improve the host protective ability of BCG by generation of strong and lasting memory response by inducing T‐central memory (TCM) cells of the Th1 and Th17 lineages.^68^ Most recently Jahagirdar PS et al coencapsulated rifampicin and curcumin in polymeric nanoparticles and used it in infected macrophages. These nanoparticles improved *M. tuberculosis* clearance from the macrophages validating that these rifampicin‐curcumin nanoparticles may be a promising therapy in future.^69^ These studies open new dimensions to implement the host‐protective role of curcumin in tuberculosis treatment.

### 3.4 Epigallocatechin‐3‐gallate

Tea is an extensively consumed drink worldwide. Among the different varieties of tea consumed, black tea and green tea possess maximum antioxidant properties.^70^ Many studies have validated the role of polyphenols in tea, in improving the levels of oxidative stress, in different disease conditions.^71^, ^72^ Epigallocatechin gallate (EGCG) is one of the principal polyphenolic compound found in the leaves of the green tea plant known as *Camellia sinensis* (Figure 4D). Green tea leaves have been reported to possess anticancer, anti‐inflammatory, and antioxidant properties.^73^ According to a study by Ahmed et al, EGCG suppresses the IL‐1 beta‐induced activity and expression of COX2 and nitric oxide synthase‐2 (NOS‐2) in human chondrocytes.^74^ Another study related to chronic kidney disease suggests that EGCG attenuates oxidative stress and inflammation via regulation of NF‐κB and Nrf2/HO‐1 signaling pathways.^75^

EGCG has shown to be quite impressive in the treatment of tuberculosis. Epidemiological evidence supports the role of tea drinking in the reduced incidences of TB. Research done on the effect of green tea polyphenols on TB suggests that regular consumption of green tea reduces the risk of active TB in infected individuals.^76^, ^77^ However, the reason for the association between tea drinking and tuberculosis is still not clear. Catechin called epigallocatechin‐3‐gallate has been mostly studied in terms of its effects on TB treatment. In murine model studies and in TB patients, green tea extract has been utilized as adjuvant therapy in tuberculosis treatment owing to its ability to reduce oxidative stress.^78^, ^79^ Green tea extract also decreases the risk of delay in sputum smear conversion in pulmonary TB patients possibly by having an impact on the integrity of the mycobacterial cell wall.^80^, ^81^ Recently Grüber et al discovered that EGCG could possibly bind to mycobacterial ATP synthase and cause the inhibitory effects such as dysregulated energy production and cell wall biosynthesis.^82^ Another study by Anand et al has revealed the effect of polyphenols derived from green tea on the TACO gene, which has been studied for its involvement in inhibition of phagosome maturation during *M. tuberculosis* infection.^83^ This study describes that epigallocatechin‐3‐gallate downregulates the transcription of TACO gene in human macrophages by inhibiting the Sp1 transcription factor, which led to inhibition of mycobacterium survival within macrophages.^83^ These studies emphasize that green tea polyphenol specifically EGCG may be used as an adjunct in the prevention of tuberculosis infection and help in reversal or reduction in the side‐effects of the strong antibiotics used in the TB treatment regime. Its role as an immunotherapeutic should also be explored so that this polyphenol can be incorporated in the treatment procedure.

### 3.5 Piperine

The pharmacological activities of black pepper (known as the king of spices) are because of the presence of various phytoconstituents in it, which confer antipathogenic properties to it. Of all the available phytoconstituents, an alkaloid, piperine is the most important (Figure 4E). Piperine is a natural compound present in *Pip*er *nigrum* and *Piper longum* and is reported to have anti‐inflammatory, antimicrobial, antifungal, antioxidant, and anticarcinogenic effects.^84^ It forms an essential component of traditional medicines for ages. Piperine has always been a center of attraction in research due to its potential of being an enhancer of bioavailability of drugs through the inhibition of CYP3A4 and human P‐glycoprotein, particularly cytochrome P450‐mediated pathways.^85^, ^86^ In tuberculosis treatment, piperine acts as an inhibitor of bacterial efflux pumps and an immunomodulatory compound.^87^ Piperine is efficient against the multidrug‐resistant strains along with eliminating drug‐sensitive strains.^88^ Chabamide is a dimer of piperine and is isolated from the stems of *P. chaba*. This dimer exhibits antituberculosis activity against *M. tuberculosis*.^89^ Piperine induces the activity of the proinflammatory Th1 response, thereby, increasing lymphocyte proliferation and increased NO secretion by the macrophages. The cotherapy of piperine and rifampicin (RIF) shows a 1.4‐0.8 log decrease in the bacterial number in the lungs of mice, which is significantly higher than RIF alone.^89^ Therefore, piperine can be administered in a combination treatment along with anti‐TB drugs owing to its upregulation of Th1 immune response, to improve the overall drug response in immune‐compromised patients.^90^ Piperine has also been studied to exhibit a strong EtBr efflux inhibitory effect in *M. smegmatis*, which possibly makes it an inhibitor of the intrinsic EP system in mycobacteria.^91^ A study by Sharma et al shows that piperine reduces the the minimum inhibitory concentration (MIC) and improves the antimicrobial action of rifampicin in all bacilli tested.^92^ In the presence of rifampicin*, M. tuberculosis* RIF‐R showed overexpression of efflux pump Rv1258c. Piperine along with rifampicin inhibits the expression of Rv1258c and thus may improve the bacterial killing efficiency of rifampicin.^92^ A recent investigation has proposed that piperine slows down *M. tuberculosis* growth through RNA polymerase inhibition, which is additional knowledge from what was previously known.^93^ Lately, the role of piperine as a bioenhancer in tuberculosis treatment is being studied upon. Risorine, a novel combination of rifampicin (200 mg), isoniazid (300 mg) along with bioenhancer piperine (10 mg), has been reported to be highly useful and safe in the treatment of TB.^94^ Risorine furnishes more rifampicin in blood compared to the gastrointestinal (GI) tract as well as maintains higher blood levels compared to the conventional rifampicin, and with a better safety profile.^95^ To put it all together, piperine has immense therapeutic applications and can be recognized as an important nutraceutical in tuberculosis treatment owing to the essential adjunct role it has shown to play during the treatment course.

### 3.6 Tetrandrine

Tetrandrine is a natural compound that is extracted from *Stephania tetrandra* root. This phytochemical, is a member of isoquinolines and a bisbenzylisoquinoline alkaloid (Figure 4F). *Stephania tetrandra* plant has been extensively used in the Chinese medicinal system since ages.^96^ Several studies have studied the efficiency of tetrandrine as an inhibitor of calcium channels and an inducer of apoptosis.^96^, ^97^ This compound has been reported to be effective in various bacterial and inflammatory health issues.^98^, ^99^, ^100^  This phytochemical also lowers the plasma glucose level by increasing glucose utilization in hepatocyte for glycogen synthesis. Although quite effective in the treatment of many diseases, its effectiveness in the treatment of tuberculosis is not very well documented. Few studies have also been conducted on the role of tetrandrine in the reversal of drug resistance in a group of both isoniazid and ethambutol‐resistant clinical strains.^98^ Tetrandrine treatment together with isoniazid or ethambutol was effective in reducing the minimum inhibitory concentration of the dual‐resistant strain from drug resistance to the sensitive level for both drugs. This study suggested that the combination therapy of tetrandrine with frontline anti‐TB drugs, isoniazid or ethambutol increased the efficacy of the drug and may help in decrease in the drug dosage, thereby minimizing the ill‐effects associated with the drug.^101^ However, since there is a lot of ambiguity in the mechanism of action of tetrandrine and its immunogenic potential is mostly unknown; more studies are needed in order to explore its maximum potential in TB cure.

### 3.7 Ursolic acid (UA) and oleanolic acid (OA)

Ursolic acid (UA) and oleanolic acid (OA) are ubiquitous triterpenoids found in many kinds of medicinal plants such as *Chamaedora tepejilote* and *Lantana hispida* (Figure 4G). More than 700 research articles have discussed its role in disease management, making it a triterpenoid of huge importance.^102^ These triterpenic acids have been commonly used in the treatment of respiratory ailments such as cough, bronchitis, colds, and pneumonia.^102^ UA and OA have several biological and pharmacological effects, including antibacterial,^103^, ^104^ antiviral,^105^ antiparasitic,^104^ antioxidant,^106^ and antitumoral activities.^107^ Recent researches have revealed the immunomodulatory and mycobactericidal effect of these triterpenoids on TB pathogenesis.^108^, ^109^ UA reportedly activated NF‐κB signaling pathway and subsequently enhanced the level of NO, ROS, and TNF‐α while reducing the level of TGF‐β1.^110^, ^111^, ^112^ The combination of UA and TB drugs has shown to display synergistic interaction in the treatment of TB.^113^ A study by Sonia López‐García et al states that OA and UA have immunomodulatory effects on *M. tuberculosis*‐infected macrophages.^113^ OA and UA reduce *M. tuberculosis* growth in macrophages by enhanced production of NO, TNF‐α, and ROS. This is also accompanied by overexpression of certain cell membrane receptors like CD36 and TGR5.^114^ These are scavenger receptor and G‐protein coupled receptors responsible for lipid accumulation and mediating bile acid synthesis. It has been reported that these triterpenes exert their antimycobacterial effects by the conversion of macrophages from M2 to M1 phenotype.^114^ Both compounds, alone and in combination, have been studied to be effective against intracellular bacteria even at low doses; with a higher expression of IFN‐γ and TNF‐α in the lungs compared to untreated control. Therefore, UA and OA have antimicrobial activity together with an immune‐stimulatory potential that can be used for the control of mycobacterial infection.^113^

### 3.8 Andrographolide

Andrographolide is a bicyclic diterpenoid compound (Figure 4H) found in *Andrographis paniculata*, which has been widely used in traditional medicines across Asian countries as an immune booster. It has antibacterial,^115^ antimalarial,^116^ analgesic,^117^ antihepatotoxic,^118^ and immunomodulatory properties.^119^ Studies in the murine model have shown that andrographolide has been effective in significantly reducing Experimental Autoimmune Encephalomyelitis (EAE) symptoms. A recent study by Liao et al has shown the potential of this diterpenoid in the treatment of steroid‐resistant airway hyperresponsiveness in patients with asthma.^120^ The immune‐stimulatory properties of *Andrographis paniculata* pave way for its role in the treatment of gastrointestinal and respiratory tract infections.^121^, ^122^ These studies make this compound very promising to be used against *M. tuberculosis*. However, very less research has been carried out globally to examine its in vitro and in vivo potency in the case of *M. tuberculosis*. Though andrographolide has shown to be cytotoxic against *M. microti*, *M. bovis*, and *M. canettii*, studies for its role in the treatment of *M. tuberculosis* are limited.^123^, ^124^ In a study by Prabhu et al, docking analysis and molecular simulation establish aminoglycoside 2‐N‐acetyltransferase (AAC) as a possible target of andrographolide in *M. tuberculosis*.^125^ AAC is an enzyme that plays a key role in acetylation of an important intermediate of mycothiol synthesis, which is a major reducing agent in mycobacteria in‐charge of regulating the cellular redox potential.^126^ Apart from this, AAC may catalyze the acetylation of the 2′ hydroxyl or amino group of a broad spectrum of aminoglycosides and thereby confer resistance to aminoglycosides.^127^ This in silico study gives us a direction to explore the vital role of andrographolide as an antimycobacterial agent that targets aminoglycoside 2‐N‐acetyltransferase in *M. tuberculosis*. As it has immunomodulatory characteristics, further in vivo studies and clinical trials are needed to substantiate its crucial role as a promising drug or adjunct in tuberculosis treatment.

### 3.9 Resveratrol

Resveratrol is a natural polyphenolic phytochemical (Stilbenoid) that is extremely enriched in red wine, the skin of grapes, peanuts, and some berries (Figure 4I). It is synthesized by the plant in response to a pathogenic attack or under stress conditions. This stilbenoid is found to be the focal point of a plethora of investigations because of its extensive use in treatment of different diseases. It has been used worldwide in the treatment of viral diseases, cancer, and neurological diseases owing to its anti‐inflammatory, antioxidant, and chemotherapeutic properties.^128^, ^129^, ^130^, ^131^ It has antimycobacterial activity against nonvirulent strains of TB such as H37Ra and BCG. Hong Yang et al have reported in an in vitro study that pretreatment with resveratrol in *M. tuberculosis*‐infected macrophages, inhibits the activation of pathways such as TAK1, MAPK, and NF‐κB and therefore resulting in the reduction in the levels of proinflammatory cytokines.^132^ Furthermore, it has been observed that mice treated with resveratrol are more resistant to *M. tuberculosis* infection compared to untreated control as they harbored less bacterial loads, and lower lung impairment as seen through histological studies.^132^ In 2019, Rosa et al developed isoniazid‐resveratrol cocrystals (INH‐RES) to increase the solubility, stability, and bioavailability of resveratrol for the treatment of cutaneous TB, locally and found it to be effective in disease cure.^133^ But, to date, we do not have much research done on the immunomodulatory and bactericidal effects of this compound. It would also be of great interest to further study the contribution of this compound in TB disease.

### 3.10 Thymoquinone

*Nigella sativa* (black cumin) is a medicinal plant having the main active compound thymoquinone (2‐isopropyl‐5‐methyl‐1, 4‐benzoquinone) (Figure 4J). This compound has potent antibacterial, anti‐inflammatory antioxidant, antimutagenic, antitumor, and hepatoprotective effects.^134^, ^135^ Thymoquinone is the main constituent of the volatile oil extracted from *Nigella sativa* plant. It has proved to restrain the development of pathogenic bacteria by inhibiting the formation of biofilms, which shelter the microorganism from harmful factors of the environment such as the host immune system and antibiotics.^136^ Besides, when used in a synergistic therapy with standard drugs, it reduces the minimum inhibitory concentration of the drugs.^137^ Additionally, it has been used as an antifungal agent against some significant parasitic pathogens including *Candida albicans*.^138^, ^139^ It has not much been used as an antimicrobial and a lot of research is needed to exploit its potential in the treatment of infectious agents. Thymoquinone is reported to have in vitro antitubercular activity and has proved to be significantly effective against drug‐resistant *M. tuberculosis* strains by inhibiting the hepatotoxic effects of anti‐TB drugs.^140^, ^141^ The study delineated antimycobacterial effects of thymoquinone, which successfully hinders the replication of *M. tuberculosis* H37Rv and the extremely drug‐resistant, XDR strain inside Raw 264.7 macrophage cell line. It also has been described to inhibit secretion of pro‐inflammatory cytokines and NO in different cell lines after bacterial infection.^142^ However, we still have very preliminary research on the use of thymoquinone as an adjunct therapy in TB, which needs to be elaborately studied in future.

### 3.11 Reserpine

Reserpine is an alkaloid that is extracted from the roots of *Rouwolfia serpentine* plant (Figure 4K). It is efficacious in the treatment of high blood pressure and other heart diseases by reducing arterial pressure. The antihypertensive effects of reserpine are due to the antinoradrenergic effects it possesses.^143^ It blocks the vesicular monoamine transporters such as catecholamines and irreversibly inhibits the internalization and stowage of dopamine in the synaptic vesicles making it very helpful in treating hypertension and psychiatric disorders.^144^, ^145^ Reserpine also has shown the ability to inhibit the formation of biofilms by inhibiting the metabolic processes of the bacteria forming the biofilm.^146^ Its role as an efflux pump inhibitor makes the bacteria susceptible to drug treatment.^147^ Additionally, it has been reported that reserpine when administered together with a standard antibiotic led to the reduction in the MIC of the antibiotic, such as tetracycline by fourfold in treatment of *B. subtilis* infection.^148^ Reserpine has shown significant effects on the pathogen's resistance to various anti‐TB drugs during mycobacterial infections. This efflux pump inhibitor increases the susceptibility of both BCG and *M. tuberculosis* strains to the drug isoniazid and also to pyrazinamide by blocking the pyrazinoic acid efflux pump.^149^, ^150^ Jaiswal et al, in 2017, have observed that in the presence of reserpine, there was a reduction in the MIC of isoniazid and it was effective against both drug‐susceptible and drug‐resistant isolates.^151^ Another study used the derivatives of reserpine in the treatment of *M. tuberculosis* owing to its extraordinary antioxidant properties.^152^ It has also been employed for the elimination of nonreplicating *M. tuberculosis* through the use of its efflux pump inhibitor action.^153^ However, there is no work done on its role as a potential immune booster. It has been reported that reserpine acts by blocking catecholamines. Grailer et al^152^ and Nguyen et al^154^, ^155^ have reported in different studies that catecholamines acts on the immune system. It acts on the host by promoting activation of M2‐like macrophage activation. Catecholamines act on both MyD88‐dependent and MyD88‐independent signaling pathways through the activation of TLRs.^154^, ^155^  There is no knowledge of how this drug acts on the host immune system. Therefore, reserpine can be categorized among the phytochemicals, which need immediate attention from the researchers as it is a natural compound with minimum or no side effects. If it can be explored as immune booster, it would probably solve the cons associated with the DOTS therapy.

### 3.12 Pasakbumin A

Pasakbumin A is a natural compound extracted from the medicinal plant, *Eurycoma longifolia*, which is a commonly used in the treatment of fever, malaria, ulcers, and TB (Figure 4L).^156^ Apart from these above‐mentioned pharmacological activities, *Eurycoma longifolia* is widely known for its anticancer potential.^157^ However, the contribution of specific compounds extracted from *E*. *longifolia* that control intracellular *M. tuberculosis* growth has not been explored adequately. Few recent studies throw light on the role of Pasakbumin A in tuberculosis as it has anti‐TB activity against the virulent *M. tuberculosis* strain. Research done till date, state that pasakbumin A works through the activation of ERK1/2‐intermediated signaling pathways and autophagy.^158^ A combination of pasakbumin A together with rifampicin has been used to clear the bacteria remarkably by high TNF‐α production and autophagy in *M. tuberculosis*‐infected macrophages.^158^ This study anticipates the imperative domain of pasakbumin, as a much potent drug that has the capacity of being used as a new drug or as an alternate treatment in TB therapy. More substantial research is required to establish it as an anti‐TB drug.

### 3.13 Gingerol

Ginger is a common plant that grows in Asia and Africa and is found abundantly in China and India (Figure 4M). It is traditionally used to treat various ailments such as headaches, colds, cough, flu, asthma, arthritis, muscular discomfort, and any sort of inflammation.^159^, ^160^ Now, scientific studies have also proven the medicinal usage of ginger to cure symptoms associated with TB.^161^, ^162^ The use of ginger in a variety of diseases is solely because of the compounds present in it, such as gingerols, shogaols, gingerdiones, gingerdiols, and paradols.^163^ As known by the available literature, garlic extracts exhibit weak antibacterial properties but it is the essential oils obtained from ginger rhizomes that are known to be significantly antibacterial. A random study conducted in pulmonary TB patients revealed that a combination therapy of ginger together with DOTS gave significantly encouraging results.^164^ A recent paper has reported that [6]‐Gingerol has immense potential to be used as an adjunct drug, along with isoniazid, an antibiotic of the DOTS regime. Gingerol showed excellent activity against drug‐resistant and dormant bacilli.^165^ Some investigators also confirm that the bioactive phytochemicals present in ginger lower the level of effective lipid mediators such as prostaglandin and leukotriene in the treated individuals via lowering the levels of 5‐lipoxygenase or prostaglandin synthase, which further leads to a decrease in the production of pro‐inflammatory cytokines highlighting its likely effectiveness in limiting the inflammation associated with tuberculosis treatment.^166^, ^167^, ^168^ The use of gingerol may help the patients avoid steroid treatments and thus their associated side effects. Therefore, it would be very intriguing to look at the immune‐modulatory consequences of including gingerol as an adjunct therapy in the TB treatment, which may add to the efficacy of the therapy.

### 3.14 Silymarin

Silymarin is the biologically active compound derived from the seeds of milk thistle plant (Figure 4N). Milk thistle has been used since ages in treating liver ailments.^169^ The flavonoid silymarin consists of three phytochemicals, silybin, silidianin, and silicristin. Of the three phytochemicals, silybin is the most important for the effectiveness of this compound in treatment of diseases.^170^ Silymarin is used in patients with a history of alcohol use and also in drug‐induced liver injuries. It is also recommended in incidences of mushroom poisoning and in chronic hepatitis B.^171^, ^172^, ^173^ Silymarin has proved to possess significant hepatoprotective activity, as revealed by various animal model studies. It has come out to be quite safe without any major side‐effects, which makes it a phytochemical with quite promising adjuvant potential to be used in anti‐TB treatment.^174^, ^175^ Researchers have established that silymarin has the potential to be used as a prospective drug to inhibit liver damage, reestablish the membrane potential and restore the function and expression levels of hepatic enzymes.^176^, ^177^ The hepatoprotective effects of silymarin have been confirmed in many studies. Nevertheless, its experimental efficacy in minimising the level of liver damage induced by ATT is a topic of conflict among the researchers. Luangchosiri and group performed clinical trial studies to demonstrate the efficacy of silymarin in treatment of active TB patients.^176^ They found that silymarin displayed a positive hepatoprotective effect after 4 weeks of drug administration. They reported that liver damage in the silymarin‐treated group was significantly lower than in the control group. However, contrary to the previous report, Marjani et al^169^ and Zhang et al^178^ did not observe any significant consequence of using silymarin in drug‐induced hepatitis patients. However, we need more research and clinical trials before we establish the role of silymarin in TB drug‐induced hepatotoxicity because of the variation in result due to limitation in sample size and variation in the population studied. Silymarin has been reported to possess significant anti‐inflammatory and immune‐modulatory capacity as well. Silymarin has been described to contribute in TB treatment by increasing the expression level of pro‐inflammatory Th‐1 cytokines. It has shown to eliminate both the MDR and drug‐sensitive *M. tuberculosis*. However, the mechanism responsible for the fundamental activity of the extract of silymarin is still not known.^179^, ^180^ The antioxidant properties associated with this compound and the research done to date make it very crucial to be studied more extensively so that its immune‐modulatory role can be further confirmed. This phytochemical demonstrates great promises and may be studied in reinfection and reactivation studies.

### 3.15 Glycyrrhizin

Glycyrrhizin is a triterpene glycoside, the major active compound present in the roots of the perennial plant *Glycyrrhiza glabra*, also commonly known as licorice (Figure 4O).^181^ Its active components are glycyrrhetinic acid, flavonoids, hydroxyl coumarins, and b‐sitosterol. *G. glabra* has been used in traditional medicines for the treatment of various diseases and is recognized since ancient times for its ethnopharmacological properties. Glycyrrhizin is most widely used in the treatment of liver problems, as an anti‐inflammatory, laxative, antidepressive, and antidiabetic and for the treatment of stomach ulcers.^182^, ^183^ This is due to the anti‐inflammatory and immune‐boosting properties of glycyrrhizin. Several investigators have documented the role of glycyrrhizin in the treatment of liver diseases such as reducing inflammation, liver fibrosis, and promoting tissue regeneration. It has been reported to possess anti‐inflammatory and antiapoptotic effects by the suppression of TNF‐α and caspase‐3.^184^ Glycyrrhizin also leads to the upregulation of proliferating cell nuclear antigen (PCNA), suggesting its role in tissue regeneration, in case of liver injury.^185^ These properties make glycyrrhizin useful as an anti‐inflammatory agent, as an antitumor treatment, and for the treatment of viral infections such as hepatitis B and SARS and in parasitic infections. Due to its use in treatment of different diseases, the researchers have started to look into the antimicrobial potential of glycyrrhizin and have evaluated its potential against both drug‐sensitive and ‐resistant strains of microorganisms.^186^ It has been found to possess significant antimicrobial properties against both Gram‐positive and Gram‐negative bacteria. It has also been used in the treatment of *H. pylori* infection and in the treatment of peptic ulcers.^186^ Glycyrrhizin has also been studied to inhibit the propagation of methicillin‐resistant *S. aureus* (MRSA) by its bacteriostatic and bactericidal activity.^186^ Recently, the role of glycyrrhizin in the treatment of intracellular pathogen, *M. tuberculosis* has been deciphered albeit to a limited extent. It has shown to reduce the MIC of the drugs used in conventional TB therapy when used in combination.^186^ However, still we do not know the mechanism employed by glycyrrhizin for its anti‐TB effectiveness. The initial results of it acting as bactericidal agent, are convincing enough to further investigate the potential of this phytochemical as an adjunct in ATT. All of these discussed compounds are summarized in Table 1.

**TABLE 1:** List of phytochemicals having antimycobacterial properties

Name of Phytochemical	Plant of origin	Mechanism of action against *M.tb*	References
Allicin	*Allium sativum* (Garlic)	Antimycobacterial, stimulates Th1 response, antihepatotoxic	30‐42
Bergenine	Different parts of a number of plants (*Shorea robusta*)	Anti‐inflammatory, induces Th1, Th17 immune response, reduces the length of treatment	43‐48
Curcumin	*Curcuma longa* (Turmeric)	Antibacterial, immunomodulatory, enhances BCG efficacy	49‐69
Epigallocatechin gallate	*Camellia sinensis* (Green Tea)	Reduces oxidative stress, impacts integrity of mycobacterial cell wall, antioxidant	70‐83
Piperine	*Piper nigrum* (Black pepper)	Antimycobacterial, NO production, stimulates Th1 response	84‐95
Tetrandrine	*Stephania tetrandra*	Reversal of drug resistance	96‐101
Ursolic acid and Oleanolic acid	*Chamaedora tepejilote, Lantana hispida*	Antimicrobial, immunomodulatory, promotes th1 response	102‐114
Andrographolide	*Andrographis paniculata*	Antihepatotoxic, antibacterial, immunomodulatory	115‐127
Resveratrol	Grapes, berries, peanuts	antibacterial, increases resistance to *M.tb* infection	128‐133
Thymoquinone	*Nigella sativa* (Black cumin)	Effective against drug‐resistant strains, hepatoprotective	134‐142
Reserpine	*Rouwolfia serpentine*	Efflux pump inhibitor, increases the susceptibility of bacteria to antibiotics	143‐155
Pasakbumin	*Eurycoma longifolia*	Autophagy inducer, antibacterial	156‐158
Gingerol.	*Zingiber officinale* (Ginger)	Anti‐inflammatory, antibacterial, antioxidant	159‐168
Silymarin	*Silybum marianum* (Milk thistle)	Hepatoprotective, anti‐inflammatory, immunomodulatory	169‐180
Glycyrrhizin	*Glycyrrhiza glabra* (Licorice)	Anti‐inflammatory, immune booster	181‐186

## 4 THE ROLE OF PHYTOCHEMICALS IN TUBERCULOSIS MANAGEMENT: AN IMMUNOLOGICAL AND HOST HEPATO‐PROTECTIVE PERSPECTIVE

Tuberculosis is a disease characterized by both microbial infection and tissue inflammation. The innate immune cells particularly the macrophages and dendritic cells encounter *M. tuberculosis* in the lung alveoli and initiate an early antimycobacterial immune response in order to prevent the progression of the disease.^187^ However, *M. tuberculosis* has evolved a number of strategies to keep the host immune system at wonder. Susceptible individuals may have suppression in the level of T helper type 1 cells (Th1) responses, which is a result of reduction in the production of IL‐12. Less Th1 response leads to reduced levels of proinflammatory cytokines and high expression levels of anti‐inflammatory cytokines.^188^ The side‐effects of DOTS therapy on the host, including the sharp decline in protective CD4+ T‐cells makes the host vulnerable to reinfection and reactivation of the disease.^189^ However, the use of anti‐inflammatory drugs along with conventional antibiotic treatment controls the inflammation associated with the disease and increases the overall effectiveness of ATT.^190^ But, because of continuous use of these anti‐inflammatory drugs or steroids the host encounters severe side‐effects; making such treatment nonadvisable for the patients. Therefore, immunologists all over the world are looking for an approach, which could lead to upregulation of Th1 immune response selectively, with the simultaneous downregulation of the Th2 immune response.^191^ Any such compound, natural, or synthetic that can act as an inducer of selective immune response is referred to as an immunomodulator.^192^ Immunomodulators alone are not capable of getting rid of the bacteria but act on the host immune system to make it more potent in eliminating the pathogen. Recently, the use of plant‐based compounds as immunomodulators has gained huge importance. A lot of research is being done in evaluating the usefulness of plants and compounds derived from them in boosting the immune system against *M. tuberculosis*. These compounds are used with the sole purpose of balancing the pro‐inflammatory cytokines and anti‐inflammatory cytokines, which is disturbed by the bacilli for its favoured survival in the host system.^192^ Of the known phytochemicals or plant secondary metabolites whose immunomodulatory properties are very well established are very few. Most of the secondary metabolites have not been tested for their immune‐boosting potential and their role in preventing the reactivation of the disease. As their role in the prevention of hepatotoxicity and improving the overall health of the patient is gaining huge attention, these compounds may in future qualify to be used in synergistic treatment approach, along with the conventional DOTS regimen for improving the overall quality of the treatment and reducing the side effects involved with the ATT.

**FIGURE 5:**
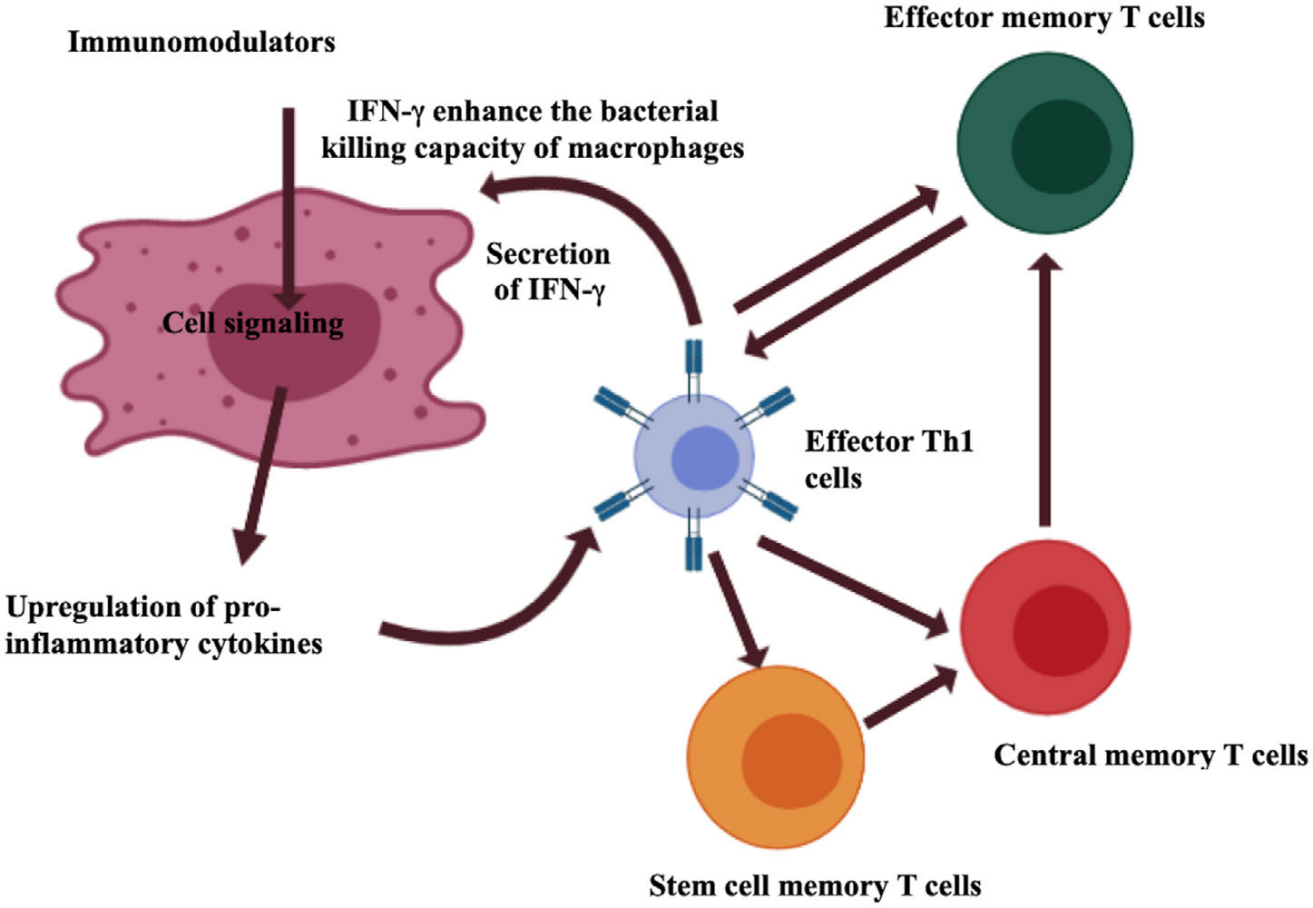
The mechanism of action of immunomodulators against TB

Alcoholic extract of *Coleus scutellarioides* (Miana leaves) induce the proinflammatory T‐lymphocyte response by increasing the levels of IFN‐γ and TNF‐α, thus acting as an immunomodulator.^192^ The killing of *M. tuberculosis* by treatment of miana extract is not due to direct inhibition of bacterial proliferation; instead, it is a result of host immunomodulation by the compound.^192^ Similarly, immunomodulatory activities of other plant secondary metabolites have been described. Garlic induces a strong protective Th1 response while also eliminating the susceptible as well as the drug‐resistant strains. Silymarin, extracted from the seeds of *Sylibym marianum*, induces a remarkable expression of Th1 immune response related cytokines both in treatment of drug‐sensitive and ‐resistant strains.^193^ Other examples of phytochemicals with reported immunomodulatory effects are piperine, an extract of chanca piedra (*Phyllanthus niruri*), extracts of Rubiaceae species, allicin from garlic, curcumin from turmeric, and gingerol from ginger. They have shown to act by restoring the Th1/Th2 balance, while acting as antioxidants, anti‐inflammatory agents, and immunomodulators and increasing the expression level of proinflammatory cytokines and NO.^194^ The mechanism of action of immunomodulators on the host system has been described in Figure 5. Phytochemicals such as curcumin may be used as an alternative to steroids used in the management of inflammation during ATT based on the finding that curcumin nanoparticles have been used restore the number and differentiation capacity of T‐cells after isoniazid treatment, both in vitro and in mice model. It has also been reported to prevent “apoptosis” in immune cells, which is stimulated by antibiotics; by the activation of the caspase‐3 pathway.^58^ Moreover, the incorporation of curcumin nanoparticles to the DOTS therapy has been studied to induce the activation and progression of TCM and thereby prevented reinfection and reactivation of the disease in mice.^195^ Administration of antibiotics during ATT leads to serious side‐effects in the host, such as hepatotoxicity and immune impairment.^195^ Certain phytochemicals due to their healing and antioxidant properties such as garlic, aqueous onion (*Alium cepa*) extract, silymarin, and nanocurcumin lead to a significant reduction in liver lesions induced by isoniazid, which has further been confirmed by the lower expression level of liver enzymes such as alanine transaminase, alkaline phosphatase, and aspartate transaminase, which indicate improvement in the condition of ailing host liver as reported in the literature available for the medicinally important plants.^196^, ^197^

Therefore, as studied for some phytochemicals, the antimycobacterial action is mainly due to their immunomodulatory properties and their ability to prevent the adverse effects of antibiotic treatment. If more such studies are conducted to further explore the bacterial clearing capacity of more phytochemicals and their mechanism of action, this may contribute significantly to the effectiveness of DOTS therapy, by reduction in the duration of TB treatment regimen, thus increasing the effective bacterial clearance and simultaneously diminishing the chances of emergence of drug resistance in the bacteria. Any contribution these phytochemicals make may add to the improvement of an existing therapy or pave way for other novel therapies. This might be a big step in eradication of TB from the population. The effect of using these plant secondary metabolites as immunomodulators or as an adjunct therapy along with the DOTS treatment has various consequences for the host.

## 5 CONCLUSION

The phytochemicals derived from plant extracts are useful not only in eliminating the bacteria but as discussed in the review may serve as prospective adjunct agents that help in reducing the after‐effects of the classical antimycobacterial drugs. These plant‐derived products assist in reinstituting the balanced level of the pro‐inflammatory and anti‐inflammatory cytokine response of the host, which is disturbed by the bacteria upon infection. Furthermore, studies on compounds with anti‐MDR‐TB and XDR‐TB activity may contribute to the management of TB very significantly as drug development against MDR‐ and XDR‐TB is the need of the hour, owing to its high prevalence and difficult management. The plant‐derived phytochemicals can also be given as personalized treatment to patients suffering from other diseases along with TB; like silymarin can be given along with DOTS to a hepatitis patient who gets infected with TB simultaneously. Many of these phytochemicals and the compounds derived from them can be administered as inhalation therapy along with DOTS to increase the effective drug concentrations within the lungs and thereby reduce the incidence of off‐target side‐effects and systemic toxicity. Although, clinical trials involving combination use of some phytochemicals with the conventional ATT have shown to be very beneficial in TB cure, but still the full potential of phytochemicals still remains undeciphered. There are several limitations, which prevent the successful use of these phytochemicals such as lack of knowledge about their interaction with normal human diet and conventional drugs and less clarity of their mechanism of action. However, these challenges could be overcome by advancing multidisciplinary and high throughput research using bioinformatics, molecular biology, and immunological approaches before it is used on animal models.

Seeing the immense potential this therapy has, it could definitely be recommended for use in the anti‐TB drug regimen in future to improve the effectiveness of the existing TB treatment procedures. In conclusion, we have presented many leads in this review article for mining of new drug candidates as boosters of host‐directed therapy against the most deadly pathogen *M. tuberculosis*.

## 6 OUTLOOK AND FUTURE PERSPECTIVES

Despite highly active research on the development of novel drugs against TBs we have quite a few drugs that have been approved for the treatment of TB in the last few decades,^198^ taking into consideration the fast emergence of drug‐resistant strains. It has been reported that the new anti‐TB drugs in clinical trials such as Sequella (SQ109), piperazine‐benzothiazinone (PBTZ169), and benzothiazinones (BTZ043) have the pharmacophore of piperine, which is a phytochemical.^199^ Therefore, a plant‐based drug molecule could be used as a research candidate for anti‐TB treatment. Isoniazid despite having enumerable side effects cannot be replaced because of its effectiveness and target binding specificity. However, any new drug that could reduce the inherent side effects of isoniazid when given in combination could revolutionize TB drug development program. Moreover, phytochemical cotherapy along with isoniazid and other anti‐TB drugs leads us toward a potent anti‐TB drug development approach and is less time and expenditure demanding compared to searching of a leading anti‐TB drug candidate. These advantages pave a promising future for the use of these phytochemicals in TB treatment.

## CONFLICT OF INTEREST

The author's confirm that there are no conflicts of interest.

## ETHICS APPROVAL

No ethical approval was required for this study.

## AUTHOR CONTRIBUTIONS

The authors confirm contribution to the paper as follows: study conception and design: Ved Prakash Dwivedi; data collection: Samreen Fatima, Anjna Kumari; analysis, interpretation, and critical revision for important intellectual content: Samreen Fatima; draft manuscript preparation: Samreen Fatima, Anjna Kumari. All authors reviewed the results and approved the final version of the manuscript.

## Data Availability

We hereby declare that this review will be openly available for all.

## References

[1] Fatima S , Kumari A , Das G , Dwivedi VP . Tuberculosis vaccine: a journey from BCG to present. Life Sci. 2020;252:117594. 32305522 10.1016/j.lfs.2020.117594

[2] World Health Organization. Global TB Report 2019 [Internet]. WHO; 2019. Available at: https://www.who.int/teams/globaltuberculosis-programme/tb-reports/globalreport2019#:~:text=The%20Global%20TB%20Report%202019,in%20202%20countries%20and%20territories.

[3] Ilievska‐Poposka B , Metodieva M , Zakoska M , et al. Latent tuberculosis infection—diagnosis and treatment. Open Access Maced J Med Sci. 2018;6(4):651‐655. 29731933 10.3889/oamjms.2018.161 PMC5927496

[4] Paul R . The threat of multidrug‐resistant tuberculosis. J Glob Infect Dis. 2018;10(3):119‐120. 30166808 10.4103/jgid.jgid_125_17 PMC6100333

[5] Forget EJ , Menzies D . Adverse reactions to first‐line antituberculosis drugs. Expert Opin Drug Saf. 2006;5(2):231‐249. 16503745 10.1517/14740338.5.2.231

[6] de Martino M , Lodi L , Galli L , Chiappini E . Immune response to *Mycobacterium tuberculosis*: a narrative review. Front Pediatr. 2019;7:350. 31508399 10.3389/fped.2019.00350 PMC6718705

[7] Saunders BM , Frank AA , Orme IM . Granuloma formation is required to contain bacillus growth and delay mortality in mice chronically infected with *Mycobacterium tuberculosis* . Immunology. 1999;98(3):324‐328. 10583589 10.1046/j.1365-2567.1999.00877.x PMC2326942

[8] Flynn JL , Chan J , Triebold KJ , et al. An essential role for interferon gamma in resistance to *Mycobacterium tuberculosis* infection. J Exp Med. 1993;178(6):2249‐2254. 7504064 10.1084/jem.178.6.2249 PMC2191274

[9] Fatima S , Kamble SS , Dwivedi VP , et al. *Mycobacterium tuberculosis* programs mesenchymal stem cells to establish dormancy and persistence. J Clin Invest. 2020;130(2):655‐661. 31647784 10.1172/JCI128043 PMC6994115

[10] Lyadova IV , Panteleev AV . Th1 and Th17 Cells in tuberculosis: protection, pathology, and biomarkers. Mediators Inflamm. 2015:854507. 26640327 10.1155/2015/854507 PMC4657112

[11] Kolls JK , Khader SA . The role of Th17 cytokines in primary mucosal immunity. Cytokine Growth Factor Rev. 2010;21(6):443‐448. 21095154 10.1016/j.cytogfr.2010.11.002 PMC3004678

[12] Barberis I , Bragazzi NL , Galluzzo L , Martini M . The history of tuberculosis: from the first historical records to the isolation of Koch's bacillus. J Prev Med Hyg. 2017;58:E9‐E12. 28515626 PMC5432783

[13] Sieniawska E , Maciejewska Turska M , Świątek L , Xiao J . Plant‐based food products for antimycobacterial therapy. eFood. 2020;1(3):199‐216.

[14] Madikizela B , Kambizi L , McGaw LJ . An ethnobotanical survey of plants used traditionally to treat tuberculosis in the eastern region of O.R. Tambo district, South Africa. S Afr J Bot. 2017;109:231‐236.

[15] Green E , Samie A , Obi CL , et al. Inhibitory properties of selected South African medicinal plants against *Mycobacterium tuberculosis* . J Ethnopharmacol. 2010;130:151‐157. 20447452 10.1016/j.jep.2010.04.033

[16] Lawal IO , Grierson DS , Afolayan AJ . Phytotherapeutic information on plants used for the treatment of tuberculosis in Eastern Cape Province, South Africa. Evid Based Complement Alternat Med. 2014;2014:735423. 24864158 10.1155/2014/735423 PMC4016884

[17] Semenya SS , Maroyi A . Ethnobotanical survey of plants used by Bapedi traditional healers to treat tuberculosis and its opportunistic infections in the Limpopo Province, South Africa. S Afr J Bot. 2019;122:401‐421.

[18] Bunalema L , Obakiro S , Tabuti JR , Waako P . Knowledge on plants used traditionally in the treatment of tuberculosis in Uganda. J Ethnopharmacol. 2014;151:999‐1004. 24365640 10.1016/j.jep.2013.12.020

[19] Nguta JM , Appiah‐Opong R , Nyarko AK , et al. Medicinal plants used to treat TB in Ghana. Int J Mycobacteriol. 2015;4:116‐123. 26972879 10.1016/j.ijmyco.2015.02.003

[20] Mohamad S , Zin NM , Wahab HA , et al. Antituberculosis potential of some ethnobotanically selected Malaysian plants. J Ethnopharmacol. 2011;133:1021‐1026. 21094237 10.1016/j.jep.2010.11.037

[21] Gupta VK , Kaushik A , Chauhan DS , Ahirwar RK , Sharma S , Bisht D . Anti‐mycobacterial activity of some medicinal plants used traditionally by tribes from Madhya Pradesh, India for treating tuberculosis related symptoms. J Ethnopharmacol. 2018;227:113‐120. 30172059 10.1016/j.jep.2018.08.031

[22] McGaw LJ , Lall N , Meyer JJ , Eloff JN . The potential of South African plants against Mycobacterium infections. J Ethnopharmacol. 2008;119:482‐500. 18805475 10.1016/j.jep.2008.08.022

[23] Chinsembu KC . Tuberculosis and nature's pharmacy of putative anti‐tuberculosisagents. Acta Trop. 2016;153:46‐56. 26464047 10.1016/j.actatropica.2015.10.004

[24] Semenya SS , Maroyi A . Medicinal plants used for the treatment of tuberculosis by Bapedi traditional healers in three districts of the Limpopo Province, South Africa. Afr J Tradit Complement Altern Med. 2013;10(2):316‐323. 24146456 10.4314/ajtcam.v10i2.17 PMC3746579

[25] Buszczyński S . Opis 125 ziół używanych w lecznictwie z podaniem ich uprawy i zastosowania. Berlin: “Przewodnik Zdrowia.” Berlin, Health Guide; 1905.

[26] Molyneux RJ , Lee ST , Gardner DR , et al. Phytochemicals: the good, the bad and the ugly? Phytochemistry. 2007;68(22‐24):2973‐2985. 17950388 10.1016/j.phytochem.2007.09.004

[27] Goldberg G . The report of a British nutrition foundation task force. Plants: Diet and Health. Oxford, London, United Kingdom: Blackwell Publishing Ltd.; 2003:347.

[28] Kerr P . Plants and tuberculosis: Phytochemicals potentially useful in the treatment of tuberculosis. Amsterdam, Netherlands: Academic Press; 2013:45‐64.

[29] Veluthoor S , Anil P , Mandal V , Mukherjee K . Phytochemicals: in pursuit of antitubercular drugs, studies in natural product chemistry. Elsevier Publ. 2012;38:417.

[30] Thomson M , Ali M . Garlic (*Allium sativum*): a review of its potential use as an anti‐cancer agent. Curr cancer drug targets. 2003;3(1):67‐81. 12570662 10.2174/1568009033333736

[31] Rivlin R S . Historical perspective on the use of garlic. J Nutr. 2001;131(3s):951S‐954S. 10.1093/jn/131.3.951S 11238795

[32] Ross ZM , O'Gara AE , Hill DJ , et al. Antimicrobial properties of garlic oil against human enteric bacteria: evaluation of methodologies and comparisons with garlic oil sulfides and garlic powder. Appl Environ Microbiol. 2001;67:475‐480. 11133485 10.1128/AEM.67.1.475-480.2001 PMC92605

[33] Bakri IM , Douglas CW . Inhibitory effect of garlic extract on oral bacteria. Arch Oral Biol. 2005;50(7):645‐651. 15892950 10.1016/j.archoralbio.2004.12.002

[34] Miron T , Rabinikov A , Mirelman D , et al. The mode of action of allicin: its ready permeability through phospholipid membranes may contribute to its biological activity. Biochim Biophys Acta. 2000;1463:20‐30. 10631291 10.1016/s0005-2736(99)00174-1

[35] Rao RR , Rao SS , et al. Inhibition of *Mycobacterium tuberculosis* by garlic extract. Nature. 1946:157:441. 10.1038/157441b0 21066575

[36] Ratnakar P , Murthy S . Purification and mechanism of antitubercular principle from garlic (*Allium sativum*) active against isoniazid susceptible and resistant *Mycobacterium tuberculosis* H37Rv. Ind J Clin Biochem. 1995;10(1):34‐38.

[37] Hasan N , Yusufb N , Toossi Z , Islam N . Suppression of *Mycobacterium tuberculosis* induced reactive oxygen species (ROS) and TNF‐α mRNA expression in human monocytes by allicin. FEBS Lett. 2006;580:2517‐2522. 16638580 10.1016/j.febslet.2006.03.071

[38] Dibua U , Odo G , Udengwu S , Esimone C . Cytotoxicity and antitubercular activity of *Allium sativum* and lantana camara against mycobacterial isolates from people living with HIV/AIDS. The Int J Inf Dis. 2010;8.1–9.

[39] Hasan N , Yusuf N , Toossi Z , Islam N . Suppression of *Mycobacterium tuberculosis* induced reactive oxygen species (ROS) and TNF‐alpha mRNA expression in human monocytes by allicin. FEBS Lett. 2006;580(10):2517‐2522. 16638580 10.1016/j.febslet.2006.03.071

[40] Viswanathan V , Phadatare AG , Mukne A . Antimycobacterial and antibacterial activity of *Allium sativum* bulbs. Ind J Pharm Sci. 2014;76(3):256‐261. PMC4090836 25035540

[41] Dwivedi VP , Bhattacharya D , Singh M , et al. Allicin enhances antimicrobial activity of macrophages during *Mycobacterium tuberculosis* infection. J Ethnopharmacol. 2019;243:111634. 30537531 10.1016/j.jep.2018.12.008

[42] Choi JA , Cho SN , Lim YJ , et al. Enhancement of the antimycobacterial activity of macrophages by ajoene. Innate Immun. 2018;24(1):79‐88. 29239661 10.1177/1753425917747975 PMC6830758

[43] Yan DB , Zhang DP , Li M , et al. Synthesis and cytotoxic activity of 3, 4, 11‐trihydroxyl modified derivatives of bergenin. Chin J Nat Med. 2014;12:929‐936. 25556064 10.1016/S1875-5364(14)60136-9

[44] Suh KS , Chon S , Choi EM . Bergenin increases osteogenic differentiation and prevents methylglyoxal‐induced cytotoxicity in MC3T3‐E1 osteoblasts. Cytotechnology. 2018;70(1):215‐224. 28895006 10.1007/s10616-017-0135-y PMC5809652

[45] Nunomura RCS , Oliveira VG , Da Silva SL , Nunomura SM . Characterization of bergenin in *Endopleura uchi* bark and its anti‐inflammatory activity. J Braz Chem Soc. 2009;20:1060‐1064.

[46] Khan H , Amin H , Ullah A , et al. Antioxidant and antiplasmodial activities of bergenin and 11‐O‐Galloylbergenin isolated from *Mallotus philippensis* . Oxid Med Cell Longev. 2016:1051925. 26998192 10.1155/2016/1051925 PMC4779831

[47] Dwivedi VP , Bhattacharya D , Yadav V , et al. The phytochemical bergenin enhances t helper 1 responses and anti‐mycobacterial immunity by activating the map kinase pathway in macrophages. Front Cell Infect Microbiol. 2017;7:149. 28507951 10.3389/fcimb.2017.00149 PMC5410567

[48] Kumar S , Sharma C , Kaushik SR , et al. The phytochemical bergenin as an adjunct immunotherapy for tuberculosis in mice. J Bio Chem. 2019;294(21):8555‐8563. 30975902 10.1074/jbc.RA119.008005 PMC6544861

[49] Boyanapalli SS , Kong AT . Curcumin, the king of spices, epigenetic regulatory mechanisms in the prevention of cancer, neurological, and inflammatory diseases. Curr Pharmacol Rep. 2015;1(2):129‐139. 26457241 10.1007/s40495-015-0018-x PMC4596544

[50] Kunnumakkara AB , Bordoloi D , Padmavathi G , et al. Curcumin, the golden nutraceutical: multitargeting for multiple chronic diseases. Br J Pharmacol. 2017;174(11):1325‐1348. 27638428 10.1111/bph.13621 PMC5429333

[51] Aggarwal BB , Sung B . Pharmacological basis for the role of curcumin in chronic diseases: an age‐old spice with modern targets. Trends Pharmacol Sci. 2009;30(2):5‐94. 10.1016/j.tips.2008.11.002 19110321

[52] Ammon HP , Wahl MA . Pharmacology of *Curcuma longa* . Planta Med. 1991;57(1):1‐7. 2062949 10.1055/s-2006-960004

[53] Ghosh D , Bagchi D , Konishi T . Clinical Aspects of Functional Foods and Nutraceuticals. Boca Raton, Florida: CRC Press (1st ed.); 2014. 10.1201/b17349.

[54] Schraufstätter E , Bernt H . Antibacterial action of curcumin and related compounds. Nature. 1949;164:456‐457. 18140450 10.1038/164456a0

[55] Gupta SC , Patchva S , Koh W , Aggarwal BB . Discovery of curcumin, a component of golden spice, and its miraculous biological activities. Cli Exp Pha Phy. 2012;39(3):283‐299. 10.1111/j.1440-1681.2011.05648.x PMC3288651 22118895

[56] Tyagi P , Singh M , Kumari H , et al. Bactericidal activity of curcumin I is associated with damaging of bacterial membrane. PLoS One. 2015;10(3):e0121313. 25811596 10.1371/journal.pone.0121313 PMC4374920

[57] Hatcher H , Planalp R , Cho J , Torti FM , Torti SV . Curcumin: from ancient medicine to current clinical trials. Cell Mol Life Sci. 2008;65(11):1631‐1652. 18324353 10.1007/s00018-008-7452-4 PMC4686230

[58] Skommer J , Wlodkowic D , Pelkonen J . Gene‐expression profiling during curcumin‐induced apoptosis reveals downregulation of CXCR4. Exp Hematol. 2007;35(1):84‐95. 17198877 10.1016/j.exphem.2006.09.006

[59] Rao CV . Regulation of COX and LOX by curcumin. Adv Exp Med Biol. 2007;595:213‐226. 17569213 10.1007/978-0-387-46401-5_9

[60] Surh YJ , Chun KS , Cha HH . Molecular mechanisms underlying chemopreventive activities of anti‐inflammatory phytochemicals: down‐regulation of COX‐2 and iNOS through suppression of NF‐kappa B activation. Mutat Res. 2001;480‐481:243‐268. 10.1016/s0027-5107(01)00183-x 11506818

[61] Jobin C , Bradham CA , Russo MP . Curcumin blocks cytokine‐mediated NF‐kappa B activation and proinflammatory gene expression by inhibiting inhibitory factor I‐kappa B kinase activity. J Immunol. 1999;163(6):3474‐3483. 10477620

[62] Mangwani N , Singh PK , Kumar V . Medicinal plants: adjunct treatment to tuberculosis chemotherapy to prevent hepatic damage. J Ayur Int Med. 2019;S0975‐9476(18):30705‐30708. 10.1016/j.jaim.2019.02.004 PMC7772497 31679802

[63] Gupta PK , Kulkarni S , Rajan R . Inhibition of intracellular survival of multi drug resistant clinical isolates of *Mycobacterium tuberculosis* in macrophages by curcumin. Op Anti Agt J. 2013;4:1‐5.

[64] Bai X , Oberley‐Deegan RE , Bai A . Curcumin enhances human macrophage control of *Mycobacterium tuberculosis* infection. Respirology. 2016;21:951‐957. 27012592 10.1111/resp.12762

[65] Shen L , Liu CC , CY A , Ji HF . How does curcumin work with poor bioavailability? Clues from experimental and theoretical studies. Sci Rep. 2016;6:20872. 26887346 10.1038/srep20872 PMC4757858

[66] Baldwin PR , Reeves AZ , Powell KR . Monocarbonyl analogs of curcumin inhibit growth of antibiotic sensitive and resistant strains of *Mycobacterium tuberculosis* . Eur J Med Chem. 2015;92:693‐699. 25618016 10.1016/j.ejmech.2015.01.020 PMC4794995

[67] Tousif S , Singh DK , Mukherjee S . Nanoparticle‐formulated curcumin prevents posttherapeutic disease reactivation and reinfection with *Mycobacterium tuberculosis* following isoniazid therapy. Front Immunol. 2017;8:739. 28713372 10.3389/fimmu.2017.00739 PMC5491555

[68] Ahmad S , Bhattacharya D , Kar S , et al. Curcumin nanoparticles enhance mycobacterium bovis bcg vaccine efficacy by modulating host immune responses. Infect Immun. 2019;87(11):e00291‐19. 10.1128/IAI.00291-19 PMC6803339 31481412

[69] Jahagirdar PS , Gupta PK , Kulkarni SP , Devarajan PV . Intramacrophage delivery of dual drug loaded nanoparticles for effective clearance of *Mycobacterium tuberculosis* . J Pharm Sci. 2020;109(7):2262‐2270. 32240695 10.1016/j.xphs.2020.03.018

[70] Leung LK , Su Y , Chen R , Zhang Z , Huang Y , Chen ZY . The aflavins in black tea and catechins in green tea are equally effective antioxidants. J Nutr. 2001;131:2248‐2251. 11533262 10.1093/jn/131.9.2248

[71] Tipoe GL , Leung TM , Hung MW , Fung ML . Green tea polyphenols as an anti‐oxidant and anti‐inflammatory agent for cardiovascular protection. Cardiovasc Hematol Disord Drug Targets. 2007;7:135‐144. 17584048 10.2174/187152907780830905

[72] Beltz LA , Bayer DK , Moss AL , Simet IM . Mechanisms of cancer prevention by green and black tea polyphenols. Anticancer Agents Med Chem. 2006;6:389‐406. 17017850 10.2174/187152006778226468

[73] Chu C , Deng J , Man Y , Qu Y . Green tea extracts epigallocatechin‐3‐gallate for different treatments. Bio Med Res int. 2017;2017:5615647. 10.1155/2017/5615647 PMC5572593 28884125

[74] Wang Y , Wang B , Du F . Epigallocatechin‐3‐Gallate attenuates oxidative stress and inflammation in obstructive nephropathy via NF‐kappaB and Nrf2/HO‐1 signalling pathway regulation. Basic Clin Pharmacol Toxicol. 2015;117:164‐172. 25625183 10.1111/bcpt.12383

[75] Ahmed S , Rahman A , Hasnain A , et al. Green tea polyphenol epigallocatechin‐3‐gallate inhibits the IL‐1 beta‐induced activity and expression of cyclooxygenase‐2 and nitric oxide synthase‐2 in human chondrocytes. Free Radic Biol Med. 2002;33:1097‐1105. 12374621 10.1016/s0891-5849(02)01004-3

[76] Chen M , Deng J , Li W . Impact of tea drinking upon tuberculosis: a neglected issue. BMC Public Health. 2015;15:515. 26021567 10.1186/s12889-015-1855-6 PMC4446809

[77] Soh AZ , Pan A , Chee C , Wang YT , et al. Tea drinking and its association with active tuberculosis incidence among middle‐aged and elderly adults: the Singapore Chinese health study. Nutrients. 2017;9(6):544. 10.3390/nu9060544 PMC5490523 28587081

[78] Guleria RS , Jain A , Tiwari V , Misra MK . Protective effect of green tea extract against the erythrocytic oxidative stress injury during *Mycobacterium tuberculosis* infection in mice. Mol Cell Biochem. 2002;236:173‐181. 12190117 10.1023/a:1016119718321

[79] Agarwal A , Prasad R , Jain A . Effect of green tea extract (catechins) in reducing oxidative stress seen in patients of pulmonary tuberculosis on DOTS Cat I regimen. Phytomedicine. 2010;17:23‐27. 19910173 10.1016/j.phymed.2009.10.019

[80] Honarvar MR , Eghtesadi S , Gill P . The effect of green tea extract supplementation on sputum smear conversion and weight changes in pulmonary TB patients: a randomized controlled trial. Med J Isl Rep Iran. 2016;30:381. PMC4972068 27493925

[81] Sun T , Qin B , Gao M . Effects of epigallocatechin gallate on the cell‐wall structure of *Mycobacterial smegmatis* mc²155. Nat Prod Res. 2015;9(22):2122‐2124. 10.1080/14786419.2014.989391 25495515

[82] Saw WG , Wu ML , Ragunathan P . Disrupting coupling within mycobacterial F‐ATP synthases subunit ε causes dysregulated energy production and cell wall biosynthesis. Sci Rep. 2019;9(1):16759. 31727946 10.1038/s41598-019-53107-3 PMC6856130

[83] Anand PK , Kaul D , Sharma M . Green tea polyphenol inhibits *Mycobacterium tuberculosis* survival within human macrophages. Int J Biochem Cell Biol. 2006;38(4):600‐609. 16352457 10.1016/j.biocel.2005.10.021

[84] Hegeto LA , Caleffi‐Ferracioli KR , Perez de Souza J . Promising antituberculosis activity of piperine combined with antimicrobials: a systematic review. Microb Drug Resist. 2019;25:120‐126. 30096263 10.1089/mdr.2018.0107

[85] Bhardwaj RK , Glaeser H , Becquemont L , et al. Piperine, a major constituent of black pepper, inhibits human P‐glycoprotein and CYP3A4. J Pharmacol Exp Ther. 2002;302:645‐650. 12130727 10.1124/jpet.102.034728

[86] Singh J , Dubey RK , Atal CK . Piperine‐mediated inhibition of glucuronidation activity in isolated epithelial cells of the guinea‐pig small intestine: evidence that piperine lowers the endogeneous UDP‐glucuronic acid content. J Pharmacol Exp Ther. 1986;236:488‐493. 3080587

[87] Singh C , Singh SK , Nath G , Rai NP . Antimycobacterial activity of *Piper longum* L. fruit extracts against multi drug resistant *Mycobacterium spp* . Phytomedicine. 2011;3:353‐361.

[88] Murase LS , Perez de Souza JV , Meneguello JE . Possible binding of piperine in *Mycobacterium tuberculosis* RNA polymerase and rifampin synergism. Antimicrob Agents Chemother. 2019;63:e02520‐18. 31481438 10.1128/AAC.02520-18 PMC6811433

[89] Sharma S , Kalia NP , Suden P , et al. Protective efficacy of piperine against *Mycobacterium tuberculosis* . Tuberculosis. 2014;94(4):389‐396. 24880706 10.1016/j.tube.2014.04.007

[90] Jin J , Zhang J , Guo N , et al. The plant alkaloid piperine as a potential inhibitor of ethidium bromide efflux in *Mycobacterium smegmatis* . J Med Micro. 2011;60(2):223‐229. 10.1099/jmm.0.025734-0 21051548

[91] Sharma S , Kumar M , Sharma S , et al. Piperine as an inhibitor of Rv1258c, a putative multidrug efflux pump of *Mycobacterium tuberculosis* . J Antimicrob Chemother. 2010;65:1694‐1701. 20525733 10.1093/jac/dkq186

[92] Raja A , Kapur A , Fijju M , SaliqueM . I . n vitro studies on efflux pump inhibition of *Catharanthus roseus* and piperine against ofloxacin resistant *M. tuberculosis* . Int J Pharma sci Inv. 2015;4(9):32‐37.

[93] Vora A , Patel S , Patel K . Role of risorine in the treatment of drug—susceptible pulmonary tuberculosis: a pilot study. J Assoc Phys India. 2016;64(11):20‐24. 27805329

[94] Patel N , Jagannath K , Vora A , Patel M , et al. A randomized, controlled, phase iii clinical trial to evaluate the efficacy and tolerability of risorine with conventional rifampicin in the treatment of newly diagnosed pulmonary tuberculosis patients. J Assoc Phys India. 2017;65(9):48‐54. 29313577

[95] Bhagya N , Chandrashekar KR . Tetrandrine—a molecule of wide bioactivity. Phytochemistry. 2016;125:5‐13. 26899361 10.1016/j.phytochem.2016.02.005

[96] Sutter MC , Wang YX . Recent cardiovascular drugs from Chinese medicinal plants. Cardiovasc Res. 1993;27:1891‐1901. 8287391 10.1093/cvr/27.11.1891

[97] Xie QM , Tang HF , Chen JQ , Bian RL . Pharmacological actions of tetrandrine in inflammatory pulmonary diseases. Acta Pharmacol Sin. 2002;23:1107‐1113. 12466048

[98] Zhang H , Gao A , Li F , et al. Mechanism of action of tetrandrine, a natural inhibitor of *Candida albicans* drug efflux pumps. Yakugaku Zasshi. 2009;129:623‐630. 19420894 10.1248/yakushi.129.623

[99] Lee YS , Han SH , Lee SH , et al. Synergistic effect of tetrandrine and ethidium bromide against methicillin‐resistant *Staphylococcus aureus* (MRSA). J Toxicol Sci. 2011;36(5):645‐651. 22008539 10.2131/jts.36.645

[100] Li XZ , Zhang L , Nikaido H . Efflux pump‐mediated intrinsic drug resistance in *Mycobacterium smegmatis* . Antimicrob Agents Chemother. 2004;48:2415‐2423. 15215089 10.1128/AAC.48.7.2415-2423.2004 PMC434187

[101] Zhang YanZJ , Xu K , et al. Tetrandrine reverses drug resistance in isoniazid and ethambutol dual drug‐resistant *Mycobacterium tuberculosis* clinical isolates. BMC Infect Dis. 2015;15:153. 25887373 10.1186/s12879-015-0905-0 PMC4417324

[102] Fontanay S , Grare M , Mayer J , et al. Ursolic, oleanolic and betulinic acids: antibacterial spectra and selectivity indexes. J Ethnopharmacol. 2008;120:272‐276. 18835348 10.1016/j.jep.2008.09.001

[103] Szakiel A , Ruszkowski D , Grudniak A , et al. Antibacterial and antiparasitic activity of oleanolic acid and its glycosides isolated from marigold (Calendula officinalis). Planta Med. 2008;74:1709‐1715. 18951335 10.1055/s-0028-1088315

[104] Balanehru S , Nagarajan B . Protective effect of oleanolic acid and ursolic acid against lipid peroxidation. Biochem Int. 1991;24(5):981‐990. 1776961

[105] You HJ , Choi CY , Kim JY , et al. Ursolic acid enhances nitric oxide and tumor necrosis‐alpha production via nuclear factor‐kappaB activation in the resting macrophages. FEBS Lett. 2001;509:156‐160. 11741581 10.1016/s0014-5793(01)03161-1

[106] Pitaloka DAE , Sukandar EY . In vitro study of ursolic acid combination first‐ line antituberculosis drugs against drug‐sensitive and drug‐resistant strains of *mycobacterium tuberculosis* . Asian J Pharm Clin Res. 2017;10(4):216–218.

[107] Liu J . Oleanolic acid and ursolic acid: research perspectives. J Ethnopharmacol. 2005;100(1‐2):92‐94. 15994040 10.1016/j.jep.2005.05.024

[108] Choi CY , You HJ , Jeong HG . Nitric oxide and tumor necrosis factor‐alfa production by oleanolic acid via nuclear factor kappaB activation in macrophages. Bioch Biophys Res Commun. 2001;288:49‐55. 10.1006/bbrc.2001.5727 11594750

[109] Murakami S , Takashima H , Sato‐Watanabe M , et al. Ursolic acid, an antagonist for transforming growth factor (TGF)‐beta1. FEBS Lett. 2004;566:55‐59. 15147868 10.1016/j.febslet.2004.04.036

[110] Yoshimura H , Sugawara K , Saito M , et al. In vitro TGF‐beta1 antagonistic activity of ursolic acid and oleanolic acid isolated from *Clerodendranthus spicatus* . Planta Med. 2003;69:673‐675. 12898427 10.1055/s-2003-41110

[111] Podder B , Jang WS , Nam KW , et al. Ursolic acid activates intracellular killing effect of macrophages during *Mycobacterium tuberculosis* infection. J Microbiol Biotechnol. 2015;25(5):738‐744. 25406534 10.4014/jmb.1407.07020

[112] Pitaloka DAE , Sukandar EY . In vitro study of ursolic acid combination first‐ line antituberculosis drugs against drug‐sensitive and drug‐resistant strains of *Mycobacterium tuberculosis* . Asian J Pharm Clin Res. 2017;10(4):216‐218.

[113] López‐García S , Castañeda‐Sanchez JI , Jiménez‐Arellanes A , et al. Macrophage activation by ursolic and oleanolic acids during mycobacterial infection. Molecules (Basel, Switzerland). 2015;20(8):14348‐14364. 10.3390/molecules200814348 PMC6332297 26287131

[114] Jiménez‐Arellanes A , Luna‐Herrera J , Cornejo‐Garrido J , et al. Ursolic and oleanolic acids as antimicrobial and immunomodulatory compounds for tuberculosis treatment. BMC Comp Alt Med. 2013;13:258. 10.1186/1472-6882-13-258 PMC3853017 24098949

[115] Mishra SK , Saangwan NS , Sangwan RS . *Andrographis paniculata*: a review. Pharmacog Rev. 2009;1:283‐298.

[116] Misra P , Pal NL , Guru PY , Katiyar JC , Srivastava V , Tandon JS . Antimalarial activity of *Andrographis paniculata* (kalmegh) against *Plasmodium berghei* NK 65 in *Mastmys natalensis* . Int J Pharmacog. 1992;30:263‐274.

[117] Madav S , Tripathi HC , Mishra SK . Analgesic, antipyretic and antiulcerogenic effect of Andrographolide. Indian J Pharm Sci. 1995;57:121‐125.

[118] Handa SS , Sharma A . Hepatoprotective activity of andrographolide from *Andrographis paniculata* against carbon tetrachloride. Indian J Med Res. 1990;92:276‐283. 2228074

[119] Burgos RA , Hidalgo MA , Carretta MD . Immunomodulatory activities induced by *Andrographis paniculata* . In: Govil JN , Singh VK , eds. Standardization of Herbal/Ayurvedic Formulations. Houston. TX: Studium Press; 2009:425‐441.

[120] Liao W , Tan WS , Wong WS . Andrographolide restores steroid sensitivity to block lipopolysaccharide/IFN‐γ‐induced IL‐27 and airway hyperresponsiveness in mice. J Immunol. 2016;196(11):4706‐4712. 27183596 10.4049/jimmunol.1502114

[121] Dhiman A , Goyal J , Sharma K , et al. A review on medicinal prospectives of *Andrographis paniculata* Nees. J Pharm Sci Innov. 2012;1(1):1‐4.

[122] Okhuarobo A , Falodun JE , Erharuyi O , et al. Harnessing the medicinal properties of *Andrographis paniculata* for diseases and beyond: a review of its phytochemistry and pharmacology. Asian Pac J Trop Dis. 2014;4:213‐222.

[123] Garg HK , Shrivastava A . Clinical use of Andrographolide as a potential drug against vole tuberculosis. J Pure Appl Zool. 2013;1(3):223‐226.

[124] Garg HK , Shrivastava A . Cytotoxic potential of Andrographolide against Bovine Tuberculosis. IOSR—JPBS. 2013;8(5):1‐4.

[125] Prabu A , Hassan S , Prabuseenivasana AS , et al. Andrographolide: a potent antituberculosis compound that targets aminoglycoside 2‐N‐ acetyltransferase in *Mycobacterium tuberculosis* . J Mol Graph Mod. 2015;61:133‐140. 10.1016/j.jmgm.2015.07.001 26245695

[126] Vetting MW , Hegde SS , Javid‐Majd F , et al. Aminoglycoside 2'‐N‐acetyltransferase from *Mycobacterium tuberculosis* in complex with coenzyme A and aminoglycoside substrates. Nat Struct Biol. 2002;9(9):653‐658. 12161746 10.1038/nsb830

[127] Aínsa JA , Pérez E , Pelicic V , et al. Aminoglycoside 2'‐N‐ acetyltransferase genes are universally present in mycobacteria: characterization of the aac(2')‐Ic gene from *Mycobacterium tuberculosis* and the aac(2')‐Id gene from *Mycobacterium smegmatis* . Mol Microbiol. 1997;24(2):431‐441. 9159528 10.1046/j.1365-2958.1997.3471717.x

[128] Jang M , Cai L , Udeani GO , et al. Cancer chemopreventive activity of resveratrol, a natural product derived from grapes. Science. 1997;275(5297):218‐220. 8985016 10.1126/science.275.5297.218

[129] Aggarwal BB , Bhardwaj A , Aggarwal RS , et al. Role of resveratrol in prevention and therapy of cancer: preclinical and clinical studies. Anticancer Res. 2004;24(5A):2783‐2840. 15517885

[130] Das S , Das DK . Anti‐inflammatory responses of resveratrol. Inflamm, Allergy Drug Targets. 2007;6:168‐173. 17897053 10.2174/187152807781696464

[131] Smolarz HD , Swatko‐Ossor M , Ginalska G , Medyńska E . Antimycobacterial effect of extract and its components from *Rheum rhaponticum* . J AOAC Int. 2013;96(1):155‐160. 23513971 10.5740/jaoacint.12-010

[132] Yang H , Hu J , Chen YJ , Ge B . Role of Sirt1 in innate immune mechanisms against *Mycobacterium tuberculosis* via the inhibition of TAK1 activation. Arch Biochem Biophy. 2019;667:49‐58. 10.1016/j.abb.2019.04.006 31029687

[133] Rosa J , Machado TC , da Silva AK , et al. Isoniazid‐resveratrol cocrystal: a novel alternative for topical treatment of cutaneous tuberculosis. ACS Pub. 2019;19(9):5029‐5036.

[134] Nili‐Ahmadabadi A , Tavakoli F , Hasanzadeh G , et al. Protective effect of pretreatment with thymoquinone against Aflatoxin B (1) induced liver toxicity in mice. Daru. 2011;19(4):282‐287. 22615670 PMC3304388

[135] Dey D , Ray R , Hazra B . Antibacterial and antitubercular activity of selected plant products against multi‐drug resistant clinical isolates. Res. 2015;1021:1014‐1021. 10.1002/ptr.5090 24318724

[136] Chaieb K , Kouidhi B , Jrah H , et al. Antibacterial activity of Thymoquinone, an active principle of *Nigella sativa* and its potency to prevent bacterial biofilm formation. BMC Complement Altern Med. 2011;11:29. 21489272 10.1186/1472-6882-11-29 PMC3095572

[137] Paster BJ , Boches SK , Galvin JL , et al. Bacterial diversity in human subgingival plaque. J Bacteriol. 2001;183(12):3770‐3783. 11371542 10.1128/JB.183.12.3770-3783.2001 PMC95255

[138] Khan MA , Owais M . Toxicity, stability and pharmacokinetics of amphotericin B in immunomodulator tuftsin‐bearing liposomes in a murine model. J Antimicrob Chemother. 2006;58:125‐132. 16709592 10.1093/jac/dkl177

[139] Khan MA , Ashfaq MK , Zuberi HS , et al. The in vivo antifungal activity of the aqueous extract from Nigella sativa seeds. Phytother Res. 2003;17(2):183‐186. 12601685 10.1002/ptr.1146

[140] Gupta R , Thakur B , Singh P , et al. Anti‐tuberculosis activity of selected medicinal plants against multi‐drug resistant *Mycobacterium tuberculosis* isolates. Indian J Med Res. 2010;131:809‐813. 20571171

[141] Dey D , Ray R , Hazra B . Antitubercular and antibacterial activity of quinonoid natural products against multi‐drug resistant clinical isolates. Phytother Res. 2014;28(7):1014‐1021. 24318724 10.1002/ptr.5090

[142] Mahmud HA , Seo H , Kim S , et al. Thymoquinone (TQ) inhibits the replication of intracellular *Mycobacterium tuberculosis* in macrophages and modulates nitric oxide production. BMC Comp Alter Med. 2017;17(1):279. 10.1186/s12906-017-1786-0 PMC5445392 28545436

[143] Shamon SD , Perez MI . Blood pressure‐lowering efficacy of reserpine for primary hypertension. Cochrane Database Syst Rev. 2016;12(12):CD007655. 27997978 10.1002/14651858.CD007655.pub3 PMC6464022

[144] Mandela P , Chandley M , Xu YY , et al. Reserpine‐induced reduction in norepinephrine transporter function requires catecholamine storage vesicles. Neurochem Int. 2010;56(6‐7):760‐767. 20176067 10.1016/j.neuint.2010.02.011 PMC2859979

[145] Iwu MM , Court WE . Root alkaloids of *Rauwolfia vomitoria* Afz. Planta Med. 1997;32:88‐99. 10.1055/s-0028-1097565 905421

[146] Parai D , Banerjee M , Dey P , Mukherjee SK . Reserpine attenuates biofilm formation and virulence of *Staphylococcus aureus* . Microb Pathog. 2020;138:103790. 31605761 10.1016/j.micpath.2019.103790

[147] Garvey MI , Piddock LJ . The efflux pump inhibitor reserpine selects multidrug‐resistant *Streptococcus pneumoniae* strains that overexpress the ABC transporters PatA and PatB. Antimicrob Agents Chemother. 2008;52(5):1677‐1685. 18362193 10.1128/AAC.01644-07 PMC2346654

[148] Neyfakh AA , Bidnenko VE , Chen LB . Efflux‐mediated multidrug resistance in *Bacillus subtilis*: similarities and dissimilarities with the mammalian system. Proc Natl Acad Sci USA. 1991;88:4781‐4785. 1675788 10.1073/pnas.88.11.4781 PMC51750

[149] Zhang Y , Scorpio A , Nikaido H , Sun Z . Role of acid pH and deficient efflux of pyrazinoic acid in unique susceptibility of *Mycobacterium tuberculosis* to pyrazinamide. J Bacteriol. 1999;181(7):2044‐2049. 10094680 10.1128/jb.181.7.2044-2049.1999 PMC93615

[150] Zhang Y , Permar S , Sun Z . Conditions that may affect the results of susceptibility testing of *Mycobacterium tuberculosis* to pyrazinamide. J Med Microbiol. 2002;51:42‐49. 11800471 10.1099/0022-1317-51-1-42

[151] Jaiswal I , Jain A , Verma SK , et al. Effect of efflux pump inhibitors on the susceptibility of *Mycobacterium tuberculosis* to isoniazid. Lung India. 2017;34(6):499‐505. 29098993 10.4103/0970-2113.217567 PMC5684805

[152] Szumowski JD , Adams KN , Edelstein PH , Ramakrishnan L . Antimicrobial efflux pumps and *Mycobacterium tuberculosis* drug tolerance: evolutionary considerations. Curr Top Microbiol Immunol. 2013;374:81‐108. 23242857 10.1007/82_2012_300 PMC3859842

[153] Mitchison DA . A new antituberculosis drug that selectively kills nonmultiplying *Mycobacterium tuberculosis* . Cell Host Microbes. 2008;3(3):122‐124. 10.1016/j.chom.2008.02.013 18329610

[154] Grailer JJ , Haggadone MD , Sarma JV , et al. Induction of M2 regulatory macrophages through the β2‐adrenergic receptor with protection during endotoxemia and acute lung injury. J Innate Immun. 2014;6(5):607‐618. 24642449 10.1159/000358524 PMC4159611

[155] Nguyen KD , Qiu Y , Cui X , et al. Alternatively activated macrophages produce catecholamines to sustain adaptive thermogenesis. Nature. 2011;480(7375):104‐108. 22101429 10.1038/nature10653 PMC3371761

[156] Rehman SU , Choe K , Yoo HH . Review on a traditional herbal medicine, *Eurycoma longifolia* jack (tongkat ali): its traditional uses, chemistry, evidence‐based pharmacology and toxicology. Molecules (Basel, Switzerland). 2016;21(3):331. 10.3390/molecules21030331 PMC6274257 26978330

[157] Thu HE , Hussain Z , Mohamed IN , Shuid A N . Eurycoma longifolia, a potential phytomedicine for the treatment of cancer: evidence of p53‐mediated apoptosis in cancerous cells. Curr Drug Targets. 2018;19(10):1109‐1126. 28721818 10.2174/1389450118666170718151913

[158] Lee HJ , Ko HJ , Kim SH , Jung YJ . Pasakbumin A controls the growth of *Mycobacterium tuberculosis* by enhancing the autophagy and production of antibacterial mediators in mouse macrophages. PLoS ONE. 2019;14(3):e0199799. 30865638 10.1371/journal.pone.0199799 PMC6415846

[159] Nair KP . Pharmacology and nutraceutical uses of ginger. Turmeric (*Curcuma longa* L.) and Ginger (*Zingiber officinale Rosc*.)—world's invaluable medicinal spices. Cham: Springer Nature; 2019:519‐539.

[160] Shahrajabian MH , Sun W , Cheng Q . Pharmacological uses and health benefits of ginger (*Zingiber officinale*) in traditional Asian and ancient Chinese medicine, and modern practice. Not Sci Biol. 2019;11:309‐319.

[161] Maroyi A . Alternative medicines for HIV/AIDS in resource‐poor settings: insight from traditional medicines use in sub‐Saharan Africa. Trop J Pharm Res. 2014;13:1527‐1536.

[162] Nguta JM , Appiah‐Opong R , Nyarko AK , et al. Antimycobacterial and cytotoxic activity of selected medicinal plant extracts. J ethno. 2016;182:10‐15. 10.1016/j.jep.2016.02.010 PMC4801013 26875647

[163] Mohd Sahardi NFN , Makpol S . Ginger (*Zingiber officinale* Roscoe) in the prevention of ageing and degenerative diseases: review of current evidence. Evid Based Complement Alternat Med. 2019:5054395. 31531114 10.1155/2019/5054395 PMC6721508

[164] Pan MH , Hsieh MC , Kuo JM , et al. 6‐Shogaol induces apoptosis in human colorectal carcinoma cells via ROS production, caspase activation, and GADD 153 expression. Mol Nutr Food Res. 2008;52:527‐537. 18384088 10.1002/mnfr.200700157

[165] Bhaskar A , Kumari A , Singh M , et al. [6]‐Gingerol exhibits potent anti‐mycobacterial and immunomodulatory activity against tuberculosis. Int Immunopharmacol. 2020;87:106809. 32693356 10.1016/j.intimp.2020.106809

[166] Kulkarni RA , Deshpande AR . Anti‐inflammatory and antioxidant effect of ginger in tuberculosis. J Complement Integr Med. 2016;13:201‐206. 27089418 10.1515/jcim-2015-0032

[167] Tjendraputra E , Tran VH , Liu‐Brennan D , et al. Effect of ginger constituents and synthetic analogues on cyclooxygenase‐2 enzyme in intact cells. Bioorg Chem. 2001;29:156‐163. 11437391 10.1006/bioo.2001.1208

[168] Verma SK , Singh M , Jain P , Bordia A . Protective effect of ginger, *Zingiber officinale* Rosc on experimental atherosclerosis in rabbits. Indian J Exp Biol. 2004;42:736‐738. 15339040

[169] Marjani M , Baghaei P , Dizaji Kazempour , et al. Evaluation of hepatoprotective effect of silymarin among under treatment tuberculosis patients: a randomized clinical trial. Iranian J Pharma Res. 2016;15(1):247‐252. PMC4986122 27610165

[170] Rodríguez‐Flores EM , Mata‐Espinosa D , Barrios‐Payan J , et al. A significant therapeutic effect of silymarin administered alone, or in combination with chemotherapy, in experimental pulmonary tuberculosis caused by drug‐ sensitive or drug‐resistant strains: in vitro and in vivo studies. PLoS One. 2019;14(5):e0217457. 31145751 10.1371/journal.pone.0217457 PMC6542514

[171] Song Z , Deaciuc I , Song M . Silymarin protects against acute ethanol‐induced hepatotoxicity in mice. Alcoholism Clin Exp Res. 2006;30(3):407‐413. 10.1111/j.1530-0277.2006.00063.x PMC4217313 16499481

[172] Comelli MC , Mengs U , Schneider C , Prosdocimi M . Toward the definition of the mechanism of action of silymarin: activities related to cellular protection from toxic damage induced by chemotherapy. Integr Cancer Ther. 2007;6(2):120‐129. 17548791 10.1177/1534735407302349

[173] Polyak SJ , Oberlies NH , Pécheur EI , et al. Silymarin for HCV infection. Antivir Ther. 2013;18(2):141‐147. 23011959 10.3851/IMP2402 PMC4076489

[174] Hogan FS , Krishnegowda NK , Mikhailova M , Kahlenberg MS . Flavonoid, silibinin, inhibits proliferation and promotes cell‐cycle arrest of human colon cancer. J Surg Res. 2007;143(1):58‐65. 17950073 10.1016/j.jss.2007.03.080

[175] Eminzade S , Uraz F , Izzettin FV . Silymarin protects liver against toxic effects of anti‐ tuberculosis drugs in experimental animals. Nutr Metab (Lond). 2008;5:18. 18601745 10.1186/1743-7075-5-18 PMC2491620

[176] Luangchosiri C , Thakkinstian A , Chitphuk S , et al. A double‐blinded randomized controlled trial of silymarin for the prevention of antituberculosis drug‐induced liver injury. BMC Complement Altern Med. 2015;15:334. 26400476 10.1186/s12906-015-0861-7 PMC4580123

[177] Diallo T , Adjobimey M , Ruslami R , et al. Safety and side effects of rifampin versus isoniazid in children. N Engl J Med. 2018;379:454‐463. 30067928 10.1056/NEJMoa1714284

[178] Zhang S , Pan H , Peng X , et al. Preventive use of a hepatoprotectant against anti‐tuberculosis drug‐induced liver injury: a randomized controlled trial. J Gastroenterol Hepatol. 2016;31:409‐416. 26243373 10.1111/jgh.13070

[179] Karimi G , Vahabzadeh M , Lari P , et al. "Silymarin", a promising pharmacological agent for treatment of diseases. Iran J Basic Med Sci. 2011;14(4):308‐317. 23492971 PMC3586829

[180] Mamalis A , Nguyen DH , Brody N , Jagdeo J . The active natural anti‐oxidant properties of chamomile, milk thistle, and halophilic bacterial components in human skin in vitro. J Drugs Dermatol. 2013;12(7):780‐784. 23884490

[181] Utsunomiya T , Kobayashi M , Pollard RB , Suzuki F . Glycyrrhizin, an active component of licorice roots, reduces morbidity and mortality of mice infected with lethal doses of influenza virus. Antimicrob Agents Chemother. 1997;41(3):551‐556. 9055991 10.1128/aac.41.3.551 PMC163749

[182] Rahnama M , Mehrabani D , Japoni S , et al. The healing effect of licorice (*Glycyrrhiza glabra*) on *Helicobacter pylori* infected peptic ulcers. J Res Med Sci. 2013;18(6):532‐533. 24250708 PMC3818629

[183] Aly AM , Al‐Alousi L , Salem HA . Licorice: a possible anti‐inflammatory and anti‐ulcer drug. AAPS Pharm Sci Tech. 2005;6(1):E74‐E82. 10.1208/pt060113 PMC2750414 16353966

[184] Tang B , Qiao H , Meng F , Sun X . Glycyrrhizin attenuates endotoxin‐ induced acute liver injury after partial hepatectomy in rats. Braz J Med Biol Res. 2007;40(12):1637‐1646. 17994167 10.1590/s0100-879x2006005000173

[185] Jalilzadeh‐Amin G , Najarnezhad V , Anassori E , et al. Antiulcer properties of *Glycyrrhiza glabra* L. extract on experimental models of gastric ulcer in mice. Iran J Pharm Res. 2015;14(4):1163‐1170. 26664383 PMC4673944

[186] Hunter RL . The pathogenesis of tuberculosis: the early infiltrate of post‐primary ( adult pulmonary) tuberculosis: a distinct disease entity. Front Immunol. 2018;9:2108. 30283448 10.3389/fimmu.2018.02108 PMC6156532

[187] Tousif S , Singh DK , Ahmad S , et al. Isoniazid induces apoptosis of activated CD4+ T cells: implications for post‐therapy tuberculosis reactivation and reinfection. J Biol Chem. 2014;289(44):30190‐30195. 25202011 10.1074/jbc.C114.598946 PMC4215201

[188] Friedman ND , McDonald AH , Robson ME , O'Brien DP . Corticosteroid use for paradoxical reactions during antibiotic treatment for *Mycobacterium ulcerans* . PLoS Negl Trop Dis. 2012;6:e1767. 23029568 10.1371/journal.pntd.0001767 PMC3459890

[189] Rahman MA , Sobia P , Dwivedi VP , et al. *Mycobacterium tuberculosis* TlyA protein negatively regulates T helper (Th) 1 and Th17 differentiation and promotes tuberculosis pathogenesis. J Bio Chem. 2015;290(23):14407‐14417. 25847237 10.1074/jbc.M115.653600 PMC4505508

[190] Pakadang SR , Wahjuni CU , Notobroto HB , et al. Immunomodulator potential of miana leaves (*Coleus scutellarioides* (L) Benth) in prevention of tuberculosis infection. Am J Microbiol Res. 2015;3:129‐134.

[191] Esmaeil N , Anaraki SB , Gharagozloo M , Moayedi B . Silymarin impacts on immune system as an immunomodulator: one key for many locks. Int Immunopharmacol. 2017;50:194‐201. 28672215 10.1016/j.intimp.2017.06.030

[192] Gupta PK , Chakraborty P , Kumar S , et al. G1‐4A, a polysaccharide from *Tinospora cordifolia* inhibits the survival of *Mycobacterium tuberculosis* by modulating host immune responses in TLR4 dependent manner. PloS one. 2016;11(5):e0154725. 27148868 10.1371/journal.pone.0154725 PMC4858241

[193] Eminzade S , Uraz F , Izzettin FV . Silymarin protects liver against toxic effects of anti‐ tuberculosis drugs in experimental animals. Nutr Metab (Lond). 2008;5:18. 18601745 10.1186/1743-7075-5-18 PMC2491620

[194] Awofeso N . Anti‐tuberculosis medication side‐effects constitute major factor for poor adherence to tuberculosis treatment. Bull World Health Organ. 2008;86(3):B‐D. 10.2471/BLT.07.043802 PMC2647396 18368191

[195] Yang TW , Park HO , Jang HN , et al. Side effects associated with the treatment of multidrug‐resistant tuberculosis at a tuberculosis referral hospital in South Korea: a retrospective study. Medicine (Baltimore). 2017;96(28):e7482. 28700490 10.1097/MD.0000000000007482 PMC5515762

[196] Ilyas U , Katare DP , Aeri V , Naseef PP . A review on hepatoprotective and immunomodulatory herbal plants. Pharmacogn Rev. 2016;10(19):66‐70. 27041876 10.4103/0973-7847.176544 PMC4791991

[197] Adnan M , Ali S , Sheikh K , Amber R . Review on antibacterial activity of Himalayan medicinal plants traditionally used to treat pneumonia and tuberculosis. J Pharm Pharmacol. 2019;71(11):1599‐1625. 31468525 10.1111/jphp.13156

[198] Koul A , Arnoult E , Lounis N , et al. The challenge of new drug discovery for tuberculosis. Nature. 2011;1469:483‐490. 10.1038/nature09657 21270886

[199] Mu Ran , Ma Tao , Zheng Purong , et al. Anti‐HIV natural product (+)‐calanolide A is active against both drugsusceptible and drug ‐resistant strains of *Mycobacterium tuberculosis* . Bioorg Med Chem. 2004;12:1199‐1207. 14980631 10.1016/j.bmc.2003.11.012

